# Preliminary Analysis and Proof-of-Concept Validation of a Neuronally Controlled Visual Assistive Device Integrating Computer Vision with EEG-Based Binary Control

**DOI:** 10.3390/s25165187

**Published:** 2025-08-21

**Authors:** Preetam Kumar Khuntia, Prajwal Sanjay Bhide, Pudureddiyur Venkataraman Manivannan

**Affiliations:** Department of Mechanical Engineering, Indian Institute of Technology Madras, Chennai 600036, India; me22s013@smail.iitm.ac.in (P.S.B.); pvm@iitm.ac.in (P.V.M.)

**Keywords:** ArUco markers, Brain–Computer interface, DH Parameters, EEG, gradient descent, visual assistive device, YOLOv8n

## Abstract

Contemporary visual assistive devices often lack immersive user experience due to passive control systems. This study introduces a neuronally controlled visual assistive device (NCVAD) that aims to assist visually impaired users in performing reach tasks with active, intuitive control. The developed NCVAD integrates computer vision, electroencephalogram (EEG) signal processing, and robotic manipulation to facilitate object detection, selection, and assistive guidance. The monocular vision-based subsystem implements the YOLOv8n algorithm to detect objects of daily use. Then, audio prompting conveys the detected objects’ information to the user, who selects their targeted object using a voluntary trigger decoded through real-time EEG classification. The target’s physical coordinates are extracted using ArUco markers, and a gradient descent-based path optimization algorithm (POA) guides a 3-DoF robotic arm to reach the target. The classification algorithm achieves over 85% precision and recall in decoding EEG data, even with coexisting physiological artifacts. Similarly, the POA achieves approximately 650 ms of actuation time with a 0.001 learning rate and 0.1 cm^2^ error threshold settings. In conclusion, the study also validates the preliminary analysis results on a working physical model and benchmarks the robotic arm’s performance against human users, establishing the proof-of-concept for future assistive technologies integrating EEG and computer vision paradigms.

## 1. Introduction

Visual perception is one of the most necessary and reliable sensory feedback mechanisms that enable a healthy human to navigate through their surrounding environment in daily life. For instance, while conducting a reach and grasp task, the visual information about the target’s positional coordinates is relayed to the person’s brain, which then processes the information to generate actuation commands to complete the task. Such a simple task, however, is a luxury for a person affected by visual impairment. For a visually challenged person, the brain has to rely on touch percepts and/or auditory feedback to try and ‘get a feel’ of the target’s whereabouts while rummaging around in their surrounding environments. This results in wasted efforts by the visually challenged individual, which might induce mental and physical fatigue. Over the past decades, a diverse range of assistive technologies has emerged, aiming to restore or augment sensory perception and support daily activities for visually impaired individuals. The following section details a literature review examining the evolution of visual assistive devices to cater to various aspects of system efficacy, starting from technical functionality to user satisfaction. Additionally, this section points out some of the primary limitations of traditional assistive technologies, and proposes the implementation of intuitive neural control technologies integrated with perception paradigms, as a means to improve the current state of visual assistive devices.

### 1.1. Literature Review on Related Work

The technology used for visual assistance can be divided into three categories: ‘vision enhancement,’ ‘vision substitution,’ and ‘vision replacement’ [1]. While ‘vision enhancement’ and ‘vision replacement’ fall primarily under clinical and surgical domains, most of the biomechanical and robotic assistive devices aim to improve the quality of life of the visually challenged by addressing problems under the ‘visual substitution’ category. These assistive devices are broadly termed visual assistive devices (VADs) [2,3,4,5,6]. One such example of smart visual assistive devices is the intelligent cane introduced in the work of Panazan and Dulf [7]. This cane is equipped with dual ultrasonic sensors, which can alert the visually challenged cane holder about detected obstacles through vibration and audio signals. The device also allows interaction with the user through switches, and its functionalities can be enhanced using a Bluetooth-connected mobile application.

However, most traditional VADs fail to attain ubiquitous acceptability primarily due to their lack of natural and intuitive user control, the devices’ failure to induce acceptable satisfaction levels, and their functional inconsistencies between controlled environments and real-life scenarios [8,9]. For instance, in their work [10], Li et al. identify the mismatches of the device to the end-user as one of the reasons for a high abandonment rate of many VADs. To ensure reliable performance in a real-life environment, robust perception systems can be integrated with contemporary assistive mechanisms. Likewise, implementing highly intuitive control mechanisms—preferably actuated by the user’s thoughts or similar neural activations—is expected to significantly improve a VAD’s corresponding user satisfaction level.

In the context of applying neural signals for the development of enabling technologies, brain–computer interfaces (BCIs) and neural prostheses have been proven to offer cutting-edge solutions [11]. BCI technology has seen diverse applications ranging from cursor control [12], wheelchair navigation [13], to robotic arm manipulation [14]. Likewise, different implantable visual neuro prostheses are developed by analyzing the information communicated among different parts of the human neuro-visual system, which aim at restoring some useful sense of vision in profoundly blind users [15,16]. However, the current state of the technology fails to offer high enough acuity towards a fully functional sense of vision, as acknowledged in the work by Eduardo Fernandez [16]. This issue may be resolved by combining perception technologies with different BCIs designed to control robotic systems that can aid human limb motion and rehabilitation [17,18,19,20].

The convergence of perception systems and BCI-based robotic assistance has introduced systems that detect event-related potentials (ERPs) such as P300 and Steady-State Visually Evoked Potentials (SSVEPs) for discrete selection tasks [13]. However, many of these systems demand sustained visual attention and are susceptible to the effect of stress [21], making them suboptimal for real-time assistive use, especially for users with visual impairment. Instead, simpler and more voluntary binary control signals such as jaw clenching and eye blinking, derived from EEG or EMG signals, have gained traction in recent studies [22,23,24,25,26]. For instance, Velez et al. [27] successfully developed an algorithm to detect and identify the artifact signals produced from jaw-clenching and eyebrow-raising gestures during EEG signal processing. To further establish the efficacy of the jaw clench-based classification models, the study by Khoshnam et al. [28] observes that the clenching is distinguished by high-frequency components which are absent during other jaw movements. The above study is designed to successfully trigger an external device using volitional contraction of jaw muscles (jaw clenching) for command signal generation.

### 1.2. Objective and Design of the Current Study

The above discussion on similar work suggests that it is possible to implement BCI-based control of external devices using simple volitional trigger commands. Likewise, it is possible to develop perception paradigm to inform its visually impaired user about the surrounding environment [29,30]. However, limited developments have successfully tailored BCI-based perception systems towards ‘vision substitution’ to enable visually impaired individuals perform real-world object manipulation tasks.

**Figure 1:**
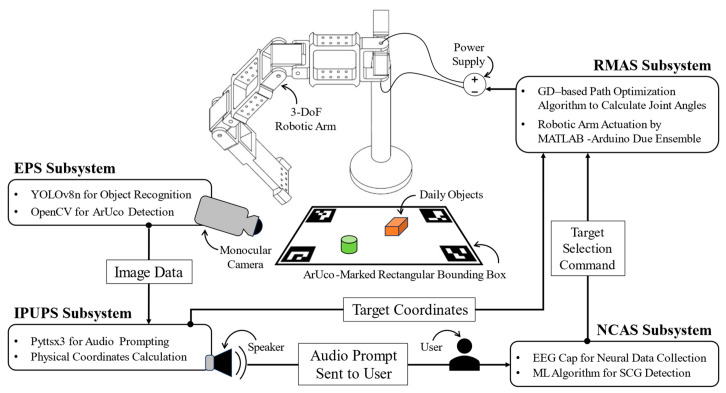
Functional block diagram representing the system architecture of the developed NCVAD and its constituent subsystems.

To bridge the gaps in contemporary research, this study introduces a Neuronally Controlled Visual Assistive Device (NCVAD), a BCI-based robotic assistive device for visually impaired individuals to conduct reach tasks in their daily lives. The NCVAD integrates electroencephalogram (EEG) signal processing with computer vision (CV) technologies to provide pertinent auditory prompt to the user, and execute a path optimization algorithm (POA) that actuates the assistive robotic arm to reach the target with improved efficacy. Additionally, the developed device incorporates a modular design and implements technologies that have been shown to work robustly irrespective of their working environment. With the aforementioned features, the NCVAD is expected to maintain its functional efficiency in practical applications while providing its users with an improved sense of control without imparting a high cognitive load on them. Figure 1 shows the developed NCVAD system with its constituents and the schema of information transfer among different subsystems.

The present approach integrates improved automation technologies and offers the proof-of-concept for a scalable and modular NCVAD system to assist visually challenged users in need. The primary novelties of the developed NCVAD can be listed as follows:

1. Utilization of a non-invasive EEG headset with 32 electrodes towards developing a fully integrated BCI and machine learning-based classification algorithm capable of detecting user-generated selection commands by analyzing concurrent physiological activities.
2. Implementation of YOLOv8n object detection algorithm along with multiple ArUco marker-based spatial transformations to facilitate target identification and real-world positional coordinate derivation in varying environments of daily usage.
3. Path planning optimization for the robotic manipulator using gradient descent (GD) technique with adjustable learning rate and error threshold values to improve efficiency and flexibility of the robot controller compared to conventional control algorithms.
4. Incorporation of modular system development methods to ensure future scalability and user-specific calibrations of the NCVAD system.
5. Evaluation of the system’s usability through real-world experimental validations and human performance assessment for temporal and behavioral analysis.

The goal of this development is to provide a comprehensive solution, not only addressing immediate accessibility challenges, but also paving the way for future applications in motor disability rehabilitation, industrial automation, and augmented neurotechnology. The following sections explain the system architecture in detail, along with how all these different subsystems work.

## 2. Materials and Methods to Describe the System Architecture

The NCVAD system developed in this work consists of four major subsystems: Environmental Perception Subsystem (EPS), Information Processing and User Prompting Subsystem (IPUPS), Neural Command Acquisition Subsystem (NCAS), and Robotic Manipulator Actuation Subsystem (RMAS). Table 1 shows different hardware components used to develop the present the NCVAD system and their corresponding technical specifications.

The EPS’s primary function is identifying the target objects and storing their respective positional information on the image plane. The IPUPS conducts the image-to-physical-world coordinate mapping activity and relays the environmental information to the user through an auditory prompt. The NCAS performs real-time neural data collection and analysis to achieve user selection detection and command transmission. Finally, the RMAS receives the target coordinates from the IPUPS and the selection command from the NCAS and then actuates the servo motor assembly to reach the target as desired by the visually challenged user. In this way, the coordinated integration of the above-mentioned subsystems is designed to establish a modular NCVAD system with real-time interactive features. This section focuses on expanding the system architecture and the protocols followed by different subsystems to conduct the desired reach-assistance activity.

### 2.1. EPS for Image Data Extraction

The primary component of the EPS is the monocular camera, which is integrated with the YOLOv8n object detection algorithm. YOLOv8 is the 8th version (v8) of the Ultralytics ‘You Only Look Once’ (YOLO) ecosystem. The YOLOv8 is widely recognized for attaining an efficient balance between accuracy and speed due to its state-of-the-art backbone and neck architectures. As per the official documentation [31], during object detection tasks, different YOLOv8 models can achieve a mean average precision (mAP) of 37.3–53.9 with an inference speed varying in the range of 80.4–479.1 ms on ONNX CPU, and 0.99–3.53 ms while running the model on an NVIDIA A100 GPU using TensorRT. With the help of the above performance parameters, the YOLOv8 models have garnered wide acceptance by many scientific studies endorsing their effectiveness and performance improvement capabilities when applied to diverse image processing and object detection tasks [32,33,34].

For our specific application of building a real-time interactive visual assistance system, the EPS must complete the object detection task with minimum latency. While ‘segmentation’ is one of the options to detect the said objects, this option requires a high floating-point operations per second (FLOPs) value to determine the exact shape and size of the targeted objects. Therefore, the current scenario necessitates the implementation of ‘object detection bounding boxes.’ This workaround facilitates the quick determination of the center of the bounding box, which, after conversion to physical coordinates, allows the robotic arm to move to a sufficiently close vicinity of the targeted object during the reach task.

**Figure 2:**
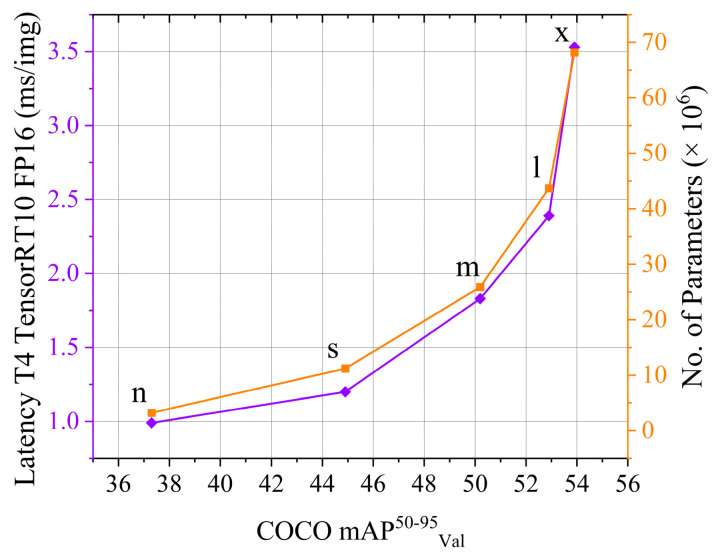
Latency and number of parameters plotted against the mAP value for different YOLOv8 models (nano, small, medium, large, extra-large) validated on the COCO dataset.

To satisfy the above requirements, the YOLOv8 models that are tuned for the bounding box generation and object detection tasks are chosen for the EPS development. Different object detection YOLOv8 models focus on optimizing different performance parameters. However, this study needs an object detection model that optimizes the inference speed to reduce the overall latency of the image data processing task. Based on the above requirements, the YOLOv8 model of ‘nano’ (n) size, i.e., YOLOv8n, is selected to develop the current EPS. The YOLOv8n model has also been proven to achieve improved precision and recall values in different research work conducted on specialized object detection tasks [35,36,37]. Figure 2 compares the performance of the YOLOv8n model to other YOLOv8 models.

**Figure 3:**
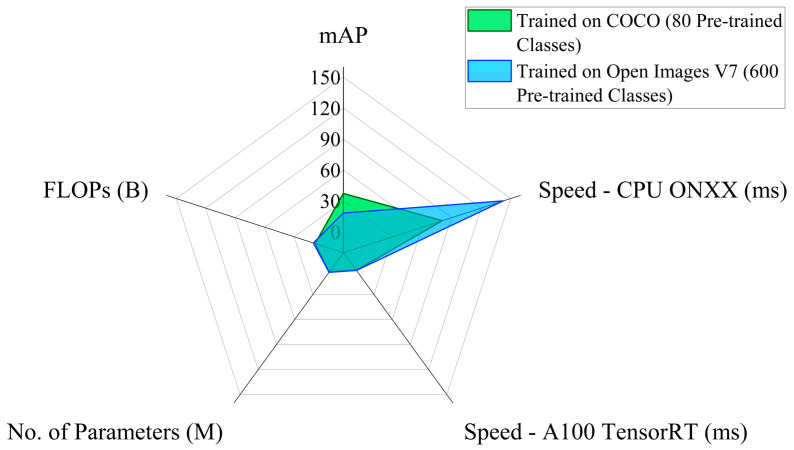
Radar plot representing the comparison between different performance parameters for YOLOv8n model trained on COCO and Open Image V7 datasets.

The YOLOv8n model implemented in the EPS is trained on the Common Objects in Context (COCO) dataset, which includes 80 pre-trained classes. COCO is a large-scale dataset extensively implemented in computer vision research and model training paradigms, primarily focusing on object detection, segmentation, key-point detection, and image captioning tasks [38,39]. While other options, such as the YOLOv8n model trained on the Open Image V7 with 600 pre-trained classes, are available, the model trained on the COCO dataset is chosen for the current study due to its improved precision, inference speed, and FLOPs values. Figure 3 summarizes the performance parameter values of the YOLOv8n model with the above two training datasets.

The object detection algorithm is integrated with the EPS by importing ‘ultralytics.YOLO’ in the Python environment and setting ‘model = YOLO (yolov8n.pt)’. The monocular camera attached to the NCVAD system is initialized using OpenCV to capture video frames. Likewise, the ‘DirectShow’ backend is implemented to improve camera compatibility on the Windows 11 PC while reducing camera access lag and latency. Upon receiving the user’s neural command to start the NCVAD system, a single frame is captured from the camera and sent to the model for prediction, identification, and bounding box generation.

### 2.2. IPUPS for Data Processing and Communication

After implementing the object detection algorithm to identify the objects in the field of view of the monocular camera, the number of identified objects and their respective class names are stored in a Python tuple. Then, the tuple is iterated sequentially to convert each string element into audible speech using the text-to-speech (TTS) library ‘pyttsx3’. This is performed with a speech rate of 200 words per minute (WPM), and a delay of 1500 ms is provided between each statement to allow the user to generate a neural selection command against their targeted object. Otherwise, they can stay idle without generating any selection commands if they do not wish to select any object. The multithreaded support of the ‘pyttsx3’ library is deployed in the NCVAD system to allow the system to execute various calculations and actuation tasks while the TTS activity is being conducted.

While the object identification information is being stored and translated to the user, the IPUPS parallelly processes the image data to generate the physical target coordinates necessary for robotic arm actuation. The EPS can approximate the target object’s position on the image plane using the centroid of its respective bounding boxes. Contrarily, for the robotic arm manipulation task, it is necessary to determine the target’s position in terms of the real-world coordinates. In a traditional setup with a stereoscopic vision camera system, the extraction of physical coordinates is achieved by triangulation and depth estimation methodologies. However, the implementation of stereo cameras is accompanied by increased cost and design complexities for the system.

In the development of the current IPUPS, a monocular camera is utilized to ensure flexibility and avoid unwanted complexity in the system design. Further, to register the actual positions of the targeted objects, it is necessary to design a system that can reliably derive the real-world coordinates of the objects from the image pixel data in various environments. In the context of image processing, Augmented Reality University of Cordoba (ArUco) markers, which are a type of binary coded fiducial markers, have proven their efficacy in various research involving tracking, navigation, and pose estimation tasks [40,41,42]. In their work on impact position determination and coordinate transformation in structural dynamics, Cepon et al. [41] demonstrate the methodology for improving the impact–pose estimation accuracy in a coordinate system using multiple ArUco markers. In another work, Xu et al. [40] present an underwater visual navigation system by placing ArUco markers in a known spatial configuration to refine pixel-to-physical coordinate transformation. A predetermined set of markers can also be deployed and calibrated in multi-marker setups to achieve more accurate overall coordinate transformation and handle occlusions and perspective distortion problems [43]. In addition, ArUco markers have also proven useful in real-time tracking applications and coordinate transformations in dynamic scenes [44].

**Figure 4:**
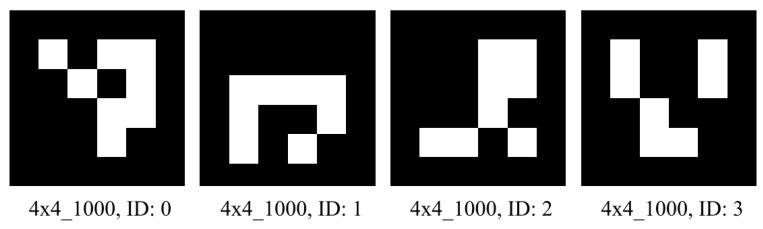
ArUco markers used in this study and their unique identifiers.

To take advantage of the flexibility and efficiency of the ArUco markers, the present study implements a muti-marker setup with four 4×4_1000 markers of size 50 mm × 50 mm for the image data processing activity while ensuring reliable data acquisition with the least computational overhead. Figure 4 depicts the four ArUco markers used to develop the IPUPS. The OpenCV (cv2) library is utilized in a Python environment to convert the captured image into grayscale and facilitate the detection of the ArUco markers using the ArucoDetector() command. This enables the pose determination of the camera with respect to the base marker (i.e., marker with ID: ‘0’) using the Perspective-n-Point (PnP) algorithm. After obtaining the rotation and transformation vectors, perspective transformation is applied to the pixel data to convert the object’s pixel coordinates into real-world coordinates, which are then stored for subsequent robotic arm manipulation tasks.

### 2.3. NCAS for Selection Command Identification

#### 2.3.1. Equipment for Data Acquisition and Processing

For the development of the NCAS, this study records neural data using a 32-channel Emotiv EPOC Flex EEG cap, which utilizes saline-soaked pads. The acquired neural data comprises time series EEG data from all the 32 channels and the frequency band power (FBP) values corresponding to each channel (five values per channel). A Lab Streaming Layer (LSL) outlet is utilized to receive the EEG data at a rate of 128 samples per second (SPS) per electrode channel. Similarly, the FBP values are collected at a rate of 8 SPS per channel. Therefore, the effective neural data recording rate becomes 8 SPS per channel when both the EEG and the FBP values are streamed simultaneously.

The EEG headset used in this study consists of Ag/AgCl electrodes with CMS/DRL referencing system, which are positioned in accordance with the international 10–20 system of placement. The Common Mode Sense (CMS) reference electrode detects the average environmental noise affecting all input electrodes (common-mode voltage/noise) and provides a baseline reference for differential amplification. Meanwhile, the said common-mode voltage/noise is actively canceled out by a proportionate feedback signal from the Driven Right Leg (DRL) reference electrode. This feedback signal is continuously applied to the user’s body, allowing the CMS/DRL referencing system to minimize ambient interference and improve the signal-to-noise ratio (SNR) of the acquired neural data.

In addition to the CMS/DRL referencing, a built-in digital fifth-order Sinc filter is implemented, which achieves band pass filtration with a bandwidth of 0.2 Hz to 45 Hz. Due to the lower cut-off frequency of this bandpass filter, the low-band interferences (signals with frequency below 0.2 Hz), such as the baseline drift and motion artifacts, are rejected from the acquired signal [45]. Similarly, the higher cut-off frequency of the Sinc filter reduces artifacts like the electromyogram (EMG) or muscle artifacts (signals with frequency above 45 Hz) in the recorded neural data [46]. Apart from this, a high attenuation notch filter at 50 Hz suppresses the power line frequencies and their associated electromagnetic harmonic components that might interfere with the EEG signal collection process.

Through the above-mentioned preprocessing steps, the acquired EEG signal is made clear of major unwanted interference without requiring further processing with dedicated spatial filtering methods [47]. In addition to the above steps, traditional approaches attempt to remove supplementary artifacts, such as the electrooculogram (EOG) signal, from the acquired neural data. Previously published studies use independent component analysis (ICA) and multiple lag regression models as some of the conventionally used tools for the EOG elimination process. This step, however, typically involves an offline postprocessing setup. The offline setup poses a significant challenge when real-time neural data acquisition and processing are essential (especially when building a user-in-the-loop BCI system). While discarding the EOG artifacts in real-time is possible, this endeavor needs specialized hardware and software components. This might result in undesired delays in signal decoding while increasing the computational overload and design complexities of the system. Therefore, to build a truly practical and efficient BCI system, it is crucial to develop neural data decoding algorithms that can function effectively even in the presence of EOG-like physiological artifacts. The following section delineates the processes followed to develop a machine learning-based classification algorithm to decode the acquired EEG signal in real-time without explicitly eliminating the coexisting physiological artifacts.

#### 2.3.2. Data Collection and Analysis for Algorithm Development

The NCAS functions by differentiating between two states of the user: i) when the user is in a relaxed state and ii) when the user is trying to generate a voluntary neural selection command. The NCAS aims to achieve this differentiation by only assessing the neural signal parameters of the user without explicitly eliminating EOG-like physiological artifacts, as discussed earlier. The current approach implements jaw clenching as the trigger activity to identify the ‘selection command generation’ (SCG) by the user. While this particular trigger is selected for the current project, the modularity of the NCVAD system allows the implementation of any such trigger activities capable of facilitating binary classification of relaxed versus selection-generation states of the user. Afterward, a neural data decoder algorithm, called the Selection Command Detection Classifier (SCDC), is developed to help the NCAS identify when the SCG activity is performed by the user.

In order to design and develop the SCDC algorithm, EEG signals were acquired from one male volunteer, 28 years of age. The neural data acquisition experiment was conducted at the VRRL Laboratory at the Indian Institute of Technology Madras, Chennai, India. The experimental procedure adheres to the Declaration of Helsinki and was approved by the ethics committee of the Indian Institute of Technology Madras, under proposal number IEC/2024-01/PVM/19 on 5 April 2024. Before the experiment began, the experimental procedures were described to the volunteer, and his prior consent was obtained. The volunteer was asked to sit comfortably in a chair wearing the Emotiv EPOC Flex EEG cap and to voluntarily clench his jaws whenever he felt comfortable. There were no further restrictions on the duration or intensity of the jaw-clenching activity to ensure the development of an intuitive, user-friendly, and versatile BCI for the NCAS. The neural datapoints against the relaxed state, as well as the jaw-clenching (selection-generation) state, were registered as a continuous data stream, and labeled as ‘0’ and ‘1’, respectively. Datapoints corresponding to a five-second duration at the beginning and the end of the data stream were eliminated to avoid the inclusion of unwarranted alterations in the acquired data. Furthermore, the labeled data samples were shuffled before training the SCDC model to ensure independently and identically distributed (IID) training samples, and prevent learning order bias by the algorithm.

**Figure 5:**
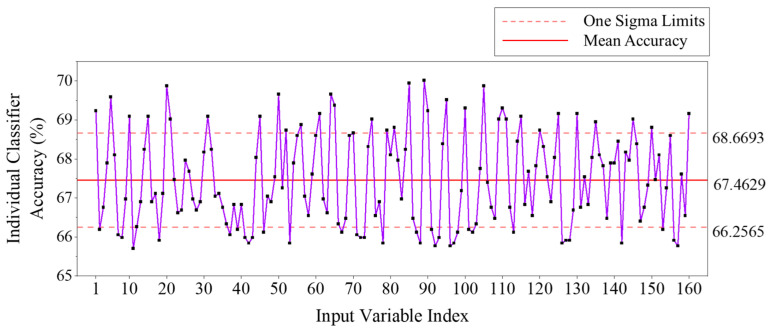
Accuracy achieved by individual FBPs during binary classification.

The collected neural data was first assessed manually to gather evidence of threshold-activation-based classification feasibility. At first glance, the FBP values corresponding to the high-beta (β2) bands of F7 and F8 electrodes seemed promising, but it was quickly noted that they resulted in false negatives roughly fifty percent of the time. In other words, around half of the time, the classifier depending only on the FBP values of F7-β2 and F8-β2 resulted in misclassifying the participant as being in the normal phase, while the participant is trying to generate a selection command by trigger activation. Therefore, all 160 FBP values were assessed for their accuracy as single classifiers to achieve the desired classification output. The performance of each single classifier was analyzed with varying upper and lower threshold values to conduct the classification task. In the end, the upper and lower thresholds providing the best performance were finalized, and the accuracy of the single classifier was recorded. As evident from Figure 5, the above analysis resulted in an achieved accuracy range of 65.70% to 70.01% for all the 160 FBPs as single classifiers.

**Figure 6:**
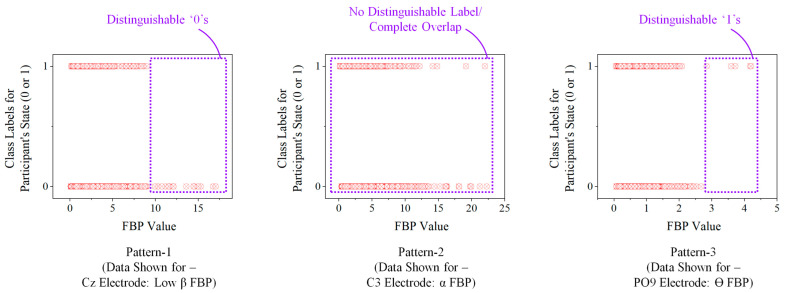
Behavioral patterns observed based on different FBP values for relaxed state (label ‘0’) and jaw-clenching state (label ‘1’) of the participant.

The above results maintain that even though it is possible to decode the neural signals for distinguishing relaxed and selection-generation phases of the volunteer, individual FBPs prove to be ‘weak learners’ for the activity. The primary reason for this is the behavioral patterns followed by the FBPs when the volunteer is in different states. Figure 6 depicts the three primary patterns observed among the collected FBP data with two different volunteer states.

Referring to the patterns shown in Figure 6, while some the FBPs show high distinguishable ‘0’ values beyond a threshold value (pattern-1), most of them have overlapping values for both ‘0’s and ‘1’s throughout the range of the FBP (pattern-2). Very few FBPs have a distinguishable ‘1’ categorization beyond a threshold or within a bounding zone (pattern-3). However, the density of datapoints and the width of the bounding zone in this case are relatively low, which makes it difficult to reduce the false negative outcomes by the predictor. This difficulty contributes to the reason why individual FBPs do not achieve acceptable classification accuracy, as evident from Figure 5 (maximum accuracy achieved: 70.01%). Nonetheless, since we have multiple weak learners, it is possible to implement ensemble learning and boosting methodologies to improve their combined prediction accuracy, especially for binary classification activities similar to the task at hand. The next section explains the development and analysis of an ensemble learning method named Adaptive Boosting (AdaBoost) deployed for the SCDC development.

#### 2.3.3. Ensemble Learning for SCDC Development

One of the primary reasons for choosing the AdaBoost algorithm is the threshold-activation-based behavior of the FBPs. In other words, the FBP values are of significance to the output prediction process only when their values either cross a threshold value or lie in a range bounded by upper and lower limits. One of the most compatible algorithms to deal with such behavior is the decision tree algorithm.

Further, the AdaBoost algorithm implements weak-learner decision trees to build a stronger classification model. The algorithm iteratively analyses individual predictors for the best classification results. Afterward, the data samples where the predictors perform poorly are given more weightage while building the next predictor. Finally, a group of predictors, each trained on the weakness of its previous tree, is generated, and the weighted output of the ensemble is taken as the final prediction of the algorithm.

**Figure 7:**
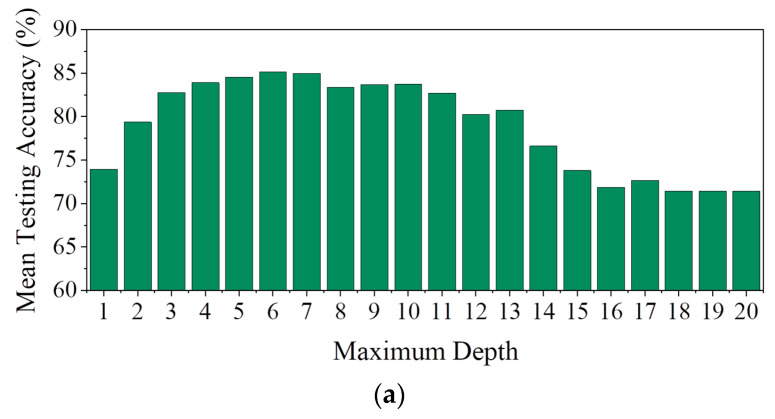

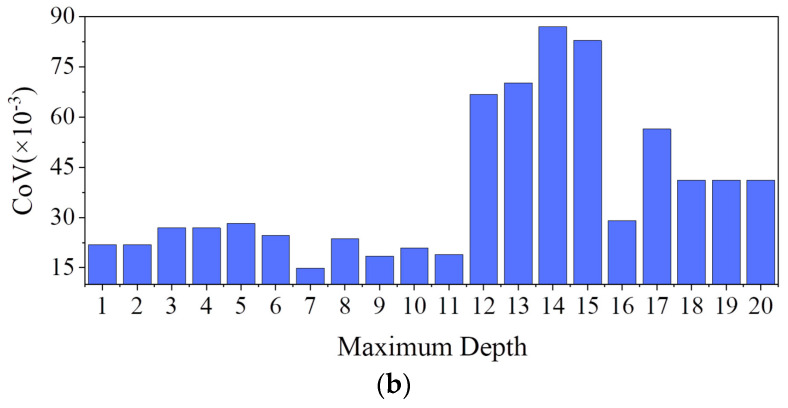
(**a**) Variation in average testing performance. (**b**) Variation in coefficient of variation (CoV) with maximum tree depth for the AdaBoost model with a fixed number of estimators and learning rate.

In order to develop the AdaBoost algorithm for the current NCAS, the neural dataset (acquired earlier) is split into training and testing subsets using the iterative five-fold cross-validation method. This splitting is achieved by first taking all the datapoints in the randomly shuffled neural data and then dividing them into five equal parts (folds) to train and test the developed SCDC model during the five iterations. In each iteration, four out of five folds (80%) of the data are used for training and optimizing the algorithm. The remaining one fold (20%) is kept aside to test the model’s performance after the training process is completed. The above steps are repeated five times in a way that each datapoint is used exactly once for the testing process (during one of the five iterations). This ensures the model’s generalizability and informs us about the model’s average performance across all five folds. In this way, the results of the five-fold cross-validation process are used to design the SCDC model that attains the best classification performance with different sets of hyperparameters. Figure 7a demonstrates the variation in the testing accuracy achieved with 300 estimators and a learning rate (LR) of one for a varying maximum depth starting from one (stumps) to twenty (decision trees).

As evident from the figure, the average accuracy across the five folds initially increases with maximum depth up to a value of six. However, increasing the maximum depth beyond seven results in a decreasing trend in accuracy. It is also observed that for a maximum depth value of six, the highest mean of 85.16% is achieved for the accuracy with a standard deviation of 2.10%. However, the lowest standard deviation of 1.26% is achieved for a tree depth of seven with a mean testing accuracy of 84.95%. Therefore, increasing the maximum depth parameter from six to seven results in a 0.25% decrease in mean testing accuracy, while the standard deviation value is improved by 40.08%. Furthermore, the model with a maximum depth of seven achieves the lowest coefficient of variation (CoV) of 0.015 (shown in Figure 7b). Therefore, a maximum depth of seven is considered as the optimal parameter for developing the AdaBoost model.

**Figure 8:**
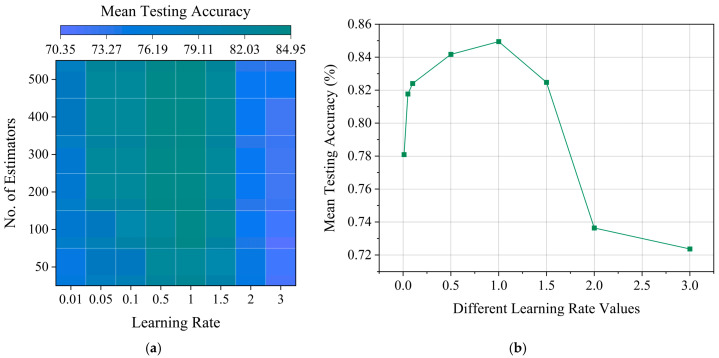
(**a**) Heatmap for variation in testing performance with different numbers of estimators and learning rate for a fixed maximum tree depth. (**b**) Variation in average testing accuracy achieved for different learning rate values with fixed tree depth and number of estimators.

The cross-validation methods are also implemented to tune the number of estimators and the LR value of the AdaBoost model to achieve the highest testing performance with a low variation in values. The testing is conducted with different sets of values for the number of estimators (50, 100, 200, 300, 400, 500) and the LR values (0.01, 0.05, 0.1, 0.5, 1, 1.5, 2, 3). Figure 8a presents the model’s performance with different hyperparameters as a heatmap for a maximum tree depth of seven. It is observed that for any number of estimators, the testing accuracy slowly increases with LR up to a value of one, before sharply falling to three (shown in Figure 8b). This suggests that with high LRs beyond one, the model fails to learn the underlying relations to generalize well, which results in a reduction in testing performance.

The highest mean testing accuracy is achieved for 300 estimators with an LR of one. While 400 estimators with one LR results in a similar mean testing accuracy, the achieved standard deviation worsens by 11.78%. Furthermore, by increasing the number of estimators from 300 to 400, the overall complexity of the model is increased with no additional improvement in performance. Therefore, 300 estimators with an LR of one and a maximum depth of seven are accepted as the optimal hyperparameters for the AdaBoost model developed as the SCDC algorithm for the NCAS.

#### 2.3.4. Algorithm Performance and Future Improvements

**Figure 9:**
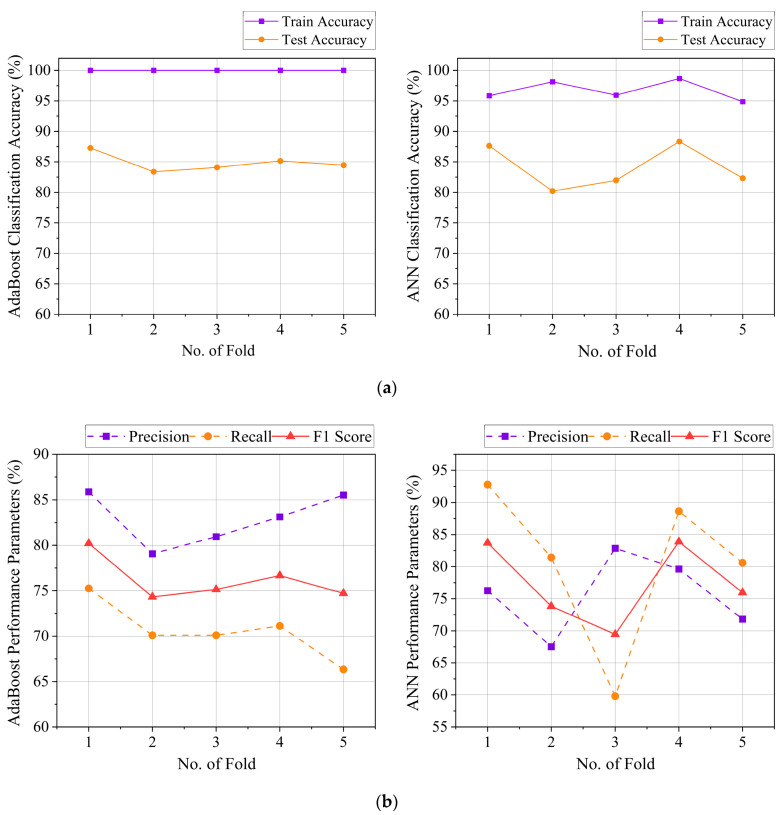
(**a**) Training and testing accuracies, and (**b**) different performance parameter values (precision, recall, and F1 score) achieved during each fold of the five-fold cross-validation process by the AdaBoost-based and the 4-layered ANN-based SCDC model.

The overall cross-validation results achieved by the developed AdaBoost algorithm are summarized in Figure 9. Apart from the accuracy levels, this study also establishes that the developed model is able to achieve maximum precision and recall values of 85.88% and 75.26%, respectively. Furthermore, the model achieves a maximum specificity level of 94.05% and an *F1 score* of 0.8022. *F1 score* is calculated as per (1), which aims to balance the model’s performance based on the harmonic mean of the precision and recall values.

(1) $$F1\;Score=2\times \frac{Precision\times Recall}{Precision+Recall}$$

In terms of true positives (TP), false positives (FP), and false negatives (FN) obtained by the model, we have [48]

(2) $$Precision=\frac{TP}{\left(TP+FP\right)}$$

(3) $$Recall=\frac{TP}{\left(TP+FN\right)}$$

Using (2) and (3), (1) can be written as

(4) $$F1\;Score=\frac{TP}{TP+\left(\frac{FP+FN}{2}\right)}$$

While real-time decoding of neural signals is challenging, especially in the presence of coexisting physiological artifacts, it is possible to improve the decoding accuracy by utilizing stronger physiological signals (e.g., replacing jaw clenching with teeth grinding in bruxism patients) and a combination of different signal extraction methodologies (e.g., EMG, ECG, SSVEP) for the classification model development [49,50,51,52,53]. The in-depth analysis of these alternate methodologies is not delineated in this study as the current goal is to validate the proof-of-concept of a modular NCVAD system, capable of interacting with a neural data acquisition modality to conduct the necessary algorithmic executions and robotic actuations. Nonetheless, this study has added a discussion on the performance of the current methodology compared to previously published research in Section 3.2 to further establish its relative efficacy.

Similar to more suitable physiological signals, the implementation of more sophisticated algorithms, such as the dense-layered Artificial Neural Network (ANN), can also improve the overall classification performance of the model [54,55]. This study has developed a 4-layered ANN model consisting of one output layer with ‘sigmoid’ activation and two hidden layers with 80 and 40 nodes, both activated by the ‘ReLU’ function. With 100 epochs and a batch size of 32 for compilation, the ANN model is able to achieve the maximum precision and recall values of 82.86% and 92.78%, respectively. This shows an increase of 23.28% in recall, while precision is reduced by 3.51%. Similarly, an increase of 4.59% in F1 score and a decrease of 0.52% in specificity are observed after implementing the developed ANN algorithm. Since the primary goal of the SCDC is to detect jaw-clenching activity labeled as ‘1’ (positive cases), it is essential to improve the recall value during the precision–recall tradeoffs, which is successfully achieved by the developed ANN model. Figure 9 demonstrates the overall training and testing performance of the developed 4-layered ANN algorithm during the five-fold cross-validation process.

To summarize the performance of the above two models, it is observed that the AdaBoost model achieves an overall accuracy of (84.88 ± 1.49)% with a precision of (82.91 ± 2.93)%, recall of (70.58 ± 3.19)%, and an F1 score of (76.21 ± 2.41)%. In comparison, the 4-layered ANN model achieves an overall accuracy of (84.09 ± 3.64)% with a precision of (75.62 ± 6.10)%, recall of (80.66 ± 12.72)%, and an F1 score of (77.37 ± 6.33)%. When the best performances of the models are considered, the maximum accuracy achieved by the ANN model is improved by 1.21% compared to that of the AdaBoost model, while the maximum training accuracy is reduced by 1.33%. The above results suggest a possible reduction in overfitting by the model. However, the ANN model suffers from higher variation in the achieved testing accuracies. A standard deviation of 3.64% is observed in the achieved testing accuracies for the ANN model, which is 2.44 times the standard deviation obtained in the testing accuracy for the AdaBoost model, while the mean value of the achieved accuracies has <1% difference between them. Furthermore, the average recall and F1 score of the ANN model are improved compared to the AdaBoost model, but this is achieved with a significantly high change in standard deviation in the corresponding values. This suggests that the developed AdaBoost model can generate more robust outputs than the ANN model, while the ANN model shows improvement in key performance parameters.

From the above discussions, it is clear that both the ANN and AdaBoost models have their own advantages and disadvantages. When the system development focuses on improved accuracy and recall value, the ANN model is more suitable. Contrarily, a system design with a preference for low deviation in the testing accuracy should prioritize the implementation of the AdaBoost model. While a Wilcoxon Signed-Rank Test (the non-parametric equivalent of paired t-test for small samples) may provide better insights into the significance level of the differences between the models’ performance, considering the observed trends and the study’s target to achieve improved precision–recall tradeoffs, the developed 4-layered ANN model is chosen due to its better comparative performance.

Furthermore, it is possible to continually improve the classifier’s performance with compatible input data and decoder algorithms in future scopes. Nonetheless, the current goal of this study is to demonstrate the feasibility of deploying an SCDC model, focusing on intuitive system development for an active voluntary command generation process. Therefore, this study implements the presently developed SCDC model to achieve the desired proof-of-concept for developing the NCAS.

#### 2.3.5. Computation Speed of the SCDC Model

After finalizing the SCDC model, it is essential to determine the time needed by the NCAS to register a selection command once the user generates it. To compute the average time, the model needs to make predictions; 10,000 sample datapoints are extrapolated from the available neural data and fed to the algorithm iteratively to generate their corresponding output class. This trial is conducted 100 times using an Intel Core i7-13650HX processor with a base clock speed of 2.6 GHz, and the time needed for each trial is recorded with a Python 3.11 interpreter. The mean time taken to make predictions for 10,000 datapoints is 0.359 s, with a sample standard deviation of 0.04 s. Therefore, it can be said with 95% confidence that the maximum time taken for 10,000 predictions will be less than 0.3684 s as per the confidence interval (CI) formula shown in (5). In other words, the NCAS will take less than 36.84µS for a single prediction for the classification task.

(5) $$CI\left(95\%\right)=µ\pm 1.96\times \frac{\sigma }{\sqrt{n}}$$

### 2.4. RMAS for Robot Path Planning and Actuation

#### 2.4.1. Robotic Arm Design

**Figure 10:**
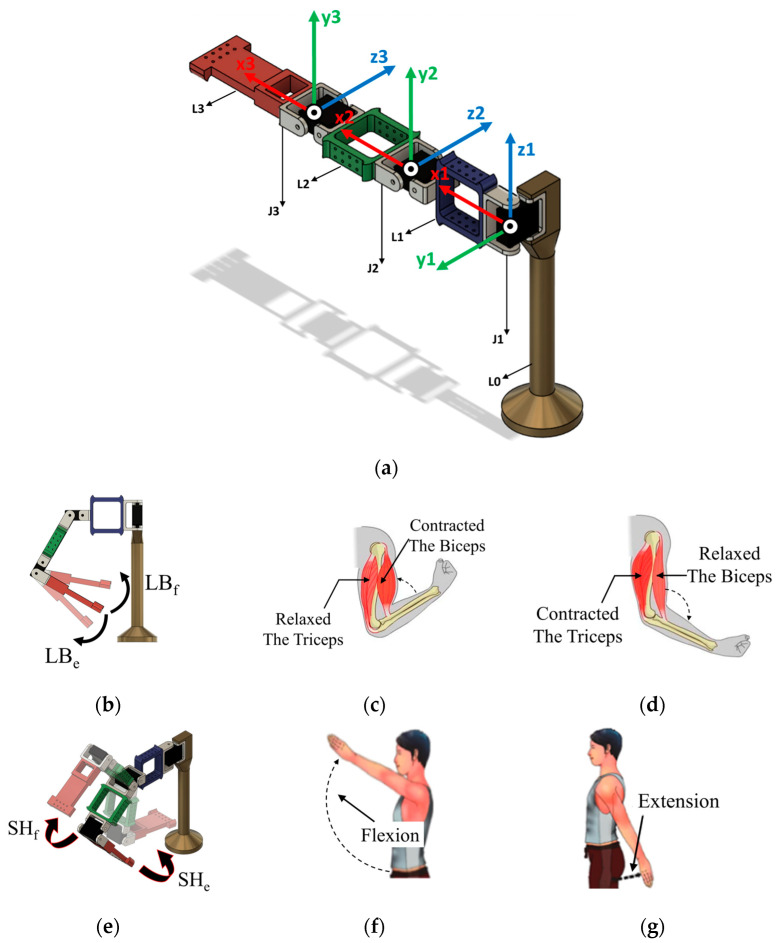

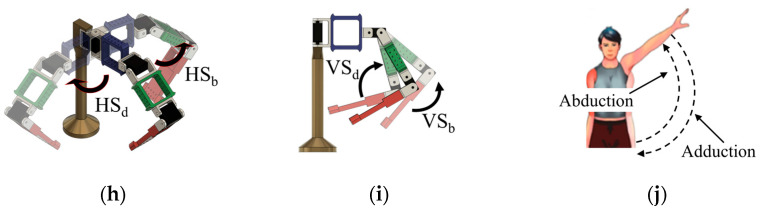
(**a**) Link and joint nomenclature and attached coordinate frames of the developed RMAS robotic manipulator (shown in isometric view). (**b**) Elbow flexion (LBf) and extension (LBe) attained by the manipulator, and its comparison to (**c**) elbow flexion and (**d**) extension performed in a human arm [56]; (**e**) shoulder flexion (SHf) and extension (SHe) achieved by the manipulator, and its comparison to (**f**) shoulder flexion and (**g**) extension movements conducted by humans [57]; (**h**) horizontal shoulder abduction (HSb) and adduction (HSd), and (**i**) vertical shoulder abduction (VSb) and adduction (VSd) performed by the RMAS manipulator, and (**j**) the corresponding human movements [57].

The Robotic Manipulator Actuation Subsystem (RMAS) is designed with a 3 Degrees-of-Freedom (DoF) robotic arm to replicate three basic human arm movements: elbow flexion/extension, shoulder flexion/extension, and shoulder abduction/adduction. To achieve the desired movements, the robotic arm setup is developed with four links, viz. L0, L1, L2, and L3, and three joint motors, viz. J1, J2, and J3, as shown in Figure 10a. Here, J3, along with L3, is responsible for replicating the elbow flexion and extension movements (Figure 10b). Likewise, J2 actuates to emulate shoulder flexion/extension (Figure 10e) and vertical shoulder abduction/adduction motions (Figure 10i). Similarly, the rotation of J1 about the vertical axis replicates the horizontal shoulder abduction/adduction motion (Figure 10h). Reference images for the elbow [56] and shoulder [57] movements of a normal human are also shown in Figure 10 to demonstrate the similarities with the discussed robotic arm movement.

**Figure 11:**
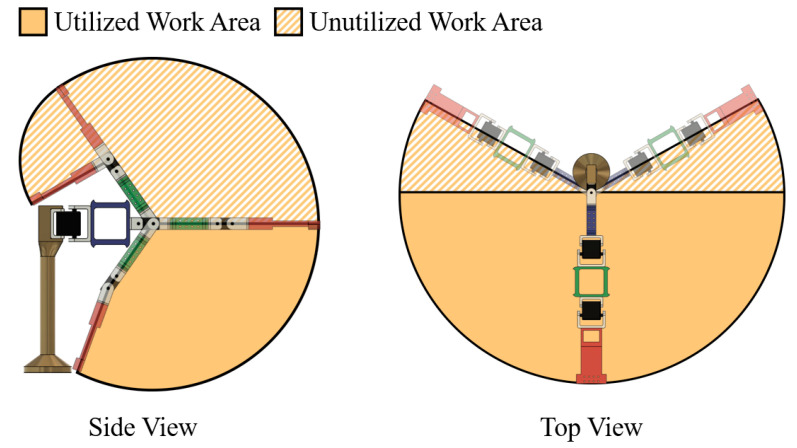
Work area of the robotic arm from side and top views, with distinction between the utilized and unutilized portions of the theoretical work area.

In the robotic arm design, L0 is fixed to the base and represents the human body. Similarly, the end effector (EE) fixed at the end of L3 represents the palm of the human body. The lengths of L2 and L3 are kept at 130 mm and 150 mm, which are approximately close to half of the average anthropometric data for the upper arm and the forearm, respectively. Further, L0 and L1 have 220 mm and 150 mm lengths, respectively, to achieve the desired work area, considering a base platform height of 25 mm. The body, links, and EE are designed and 3D-printed to achieve a proportionally equivalent work area as a natural human arm, shown in Figure 11. However, only a portion of the total theoretical work area is utilized to cater to the particular target of the NCVAD system, which is to reach targeted objects kept on a tabletop. Three RDS3115-MG motors (Robu.in, Maharashtra, India) are used to drive joints J1, J2, and J3, with a working voltage rating of 4.8–7.2 VDC. The stall torque of the motors is rated at 15 kg-cm at a voltage of 6 V, and the maximum stall torque rating is 17 kg-cm at 7.2 V. The motor models are chosen based on their ability to deliver the required torque and to ensure repeatability for conducting the reach task multiple times. MATLAB R2024b is used to implement the POA, control the motor actuation through Arduino Due microcontroller, and determine the execution time of the algorithm.

#### 2.4.2. Forward Kinematics and Control Algorithm

Appropriate coordinate frames are attached to the joints to derive the kinematic relationship between different parts of the robotic arm, as shown in Figure 10a. Further, following the conventions of forward kinematics, the Denavit–Hartenberg (DH) parameters [58] for the robotic arm are derived. Here, Ji represents the ith joint of the arm, *θ* stands for joint angle, *α* is twist angle, *d* is called link offset, and *a_i_* is link length of link Li. Furthermore, since the EE is fixed with L3, it has the same kinematic parameters derived for L3 connected to J3. Table 2 shows the associated DH parameters for the robotic arm.

By using the above DH parameters, the relevant transformation matrices for different joints are derived in the following section by implementing the homogeneous transformation matrix formula shown in (6).

(6) $$T_{n-1}^{n}=\left[\begin{array}{cccc} C_{n} & -S_{n}\left(cos\alpha_{n}\right) & S_{n}\left(sin\alpha_{n}\right) & a_{n}C_{n}\\ S_{n} & C_{n}\left(cos\alpha_{n}\right) & -C_{n}\left(sin\alpha_{n}\right) & a_{n}S_{n}\\ 0 & sin\alpha_{n} & cos\alpha_{n} & d_{n}\\ 0 & 0 & 0 & 1 \end{array}\right]$$

Here, C_i_ and *S_i_* represent *cos* (*θ_i_*) and *sin* (*θ_i_*), respectively. Likewise, *C_ij_* and *S_ij_* stand for *cos* (*θ_i_ + θ_j_*) and *sin* (*θ_i_ + θ_j_*), respectively. Following the above formula, the transformation matrices for the 1st, 2nd, and 3rd joints are

(7) $$T_{0}^{1}=\left[\begin{array}{cccc} C_{1} & 0 & S_{1} & a_{1}C_{1}\\ S_{1} & 0 & -C_{1} & a_{1}S_{1}\\ 0 & 1 & 0 & a_{0}\\ 0 & 0 & 0 & 1 \end{array}\right]$$

(8) $$T_{1}^{2}=\left[\begin{array}{cccc} C_{2} & -S_{2} & 0 & a_{2}C_{2}\\ S_{2} & C_{2} & 0 & a_{2}S_{2}\\ 0 & 0 & 1 & 0\\ 0 & 0 & 0 & 1 \end{array}\right]$$

(9) $$T_{2}^{3}=\left[\begin{array}{cccc} C_{3} & -S_{3} & 0 & a_{3}C_{3}\\ S_{3} & C_{3} & 0 & a_{3}S_{3}\\ 0 & 0 & 1 & 0\\ 0 & 0 & 0 & 1 \end{array}\right]$$

Using (7) and (8) the transformation matrix for the 2nd joint with respect to the base is derived as

(10) $$T_{0}^{2}=\left[\begin{array}{cccc} C_{1}C_{2} & -C_{1}S_{2} & S_{1} & a_{1}C_{1}+a_{2}C_{1}C_{2}\\ S_{1}C_{2} & -S_{1}S_{2} & -C_{1} & a_{1}S_{1}+a_{2}S_{1}C_{2}\\ S_{2} & C_{2} & 0 & a_{0}+a_{2}S_{2}\\ 0 & 0 & 0 & 1 \end{array}\right]$$

Similarly, the transformation matrix for the last joint as well as the EE is derived using (10) and (9) as

(11) $$T_{0}^{3}=\left[\begin{array}{cccc} C_{1}C_{23} & -C_{1}S_{23} & S_{1} & a_{1}C_{1}+a_{2}C_{1}C_{2}+a_{3}C_{1}C_{23}\\ S_{1}C_{23} & -S_{1}S_{23} & -C_{1} & a_{1}S_{1}+a_{2}S_{1}C_{2}+a_{3}S_{1}C_{23}\\ S_{23} & C_{23} & 0 & a_{0}+a_{2}S_{2}+a_{3}S_{23}\\ 0 & 0 & 0 & 1 \end{array}\right]$$

Based on the derived forward kinematic (FK) Equations (7), (10) and (11) for the three different joint actuators of the robotic arm, a control algorithm (POA) to actuate the three joints is developed by implementing the gradient descent (GD) technique. The last column of $T_{0}^{3}$, shown above in (11), represent the x, y, and z positional coordinates of the EE, represented by P_x_, P_y_, and P_z_. The GD-based optimization technique for the POA aims to minimize loss function (*Є*) iteratively until the EE reaches the positional coordinates of the targeted object. The advantage of the GD-based optimization algorithm is that it can provide faster convergence due to nonlinear increment steps for most kinematic arrangements. Therefore, it obviates the need for inverse kinematics (IK) calculations and avoids singularity issues associated with the IK process, as delineated in our previous work [59]. This work explains the IK calculations for a 3-DoF robotic arm, and derives an analytical solution for the final joint actuation angles by solving the following equations:

(12) $$\theta_{1}=\tan^{-1}\left(\frac{Y_{EED}-Y_{0}}{Z_{EED}-Z_{0}}\right)$$

(13) $$\theta_{3}=\cos^{-1}\left(\frac{{\left(X_{EED}-X_{0}\right)}^{2}+{\left(\frac{Y_{EED}-Y_{0}}{\sin\theta_{1}}\right)}^{2}-a_{1}^{2}-a_{2}^{2}}{2a_{1}a_{2}}\right)$$

(14) $$\theta_{2}=a_{1}\cos\theta_{2}+a_{2}cos\left(\theta_{2}+\theta_{3}\right)$$

Here, the terms X_EED_, Y_EED,_ and Z_EED_ represent the desired target coordinates of the EE, and X_0_, Y_0_, Z_0_ represent the x, y, and z coordinates of the origin connected to base L0. It should be noted that some of the symbols, e.g., X_EED_, Y_0_, etc. are kept different from the current convention to avoid confusion between IK calculations and GD-based optimization process, and to be consistent with the conventions followed in the cited work.

In the present RMAS design, *Є* represents the positional difference between the current and targeted EE positions. Therefore, by minimizing *Є*, the POA drives the joint motors to achieve angular positions that satisfy the FK conditions necessary to reach the targeted EE coordinates. The value of *Є* is calculated by the sum of squared error (SSE) of the current and target positional coordinates of the EE along the x, y, and z axes of the robotic arm setup, as shown in (15).

(15) $$Є=\underset{i\;\in \left\{x,\;y,\;z\right\}}{\sum }{\left(P_{i,\;\;target}-P_{i,\;\;current}\right)}^{2}$$

As discussed earlier, here P_x_, P_y_, and P_z_ represent the EE positional coordinates along the x, y, and z axes, respectively. In the above equation, the squared error is taken to ensure that the GD technique gives equal weightage to positive and negative errors during the optimization process. As per GD, to minimize *Є*, its partial differentiations with respect to the independent variables (here *θ_i_*) are calculated and multiplied with a learning rate (*η*). This generates the term (*η×∇Є*), which is then subtracted from the corresponding old values of the independent variables to update them for the next iteration.

(16) $$\left[\begin{array}{c} \theta_{1,new}\\ \theta_{2,new}\\ \vdots \\ \theta_{n,new} \end{array}\right]=\left[\begin{array}{c} \theta_{1,old}\\ \theta_{2,old}\\ \vdots \\ \theta_{n,old} \end{array}\right]-\eta \left[\begin{array}{c} \frac{\partial Є}{\partial \theta_{1}}\\ \frac{\partial Є}{\partial \theta_{2}}\\ \vdots \\ \frac{\partial Є}{\partial \theta_{n}} \end{array}\right]$$

Equation (16) is used to continue the iterative optimization process until the SSE value crosses below a predefined threshold, signifying the positional proximity of the current EE position to the targeted EE position. As discussed earlier, the GD technique changes the values of *θ_i_* at every iteration based on the instantaneous slope of the SSE vs. *θ_i_* curves. Therefore, when the slope becomes close to zero for each *θ_i_*, the control algorithm considers that *θ_i_* value as a solution to minimizing the SSE value. Based on the transformation matrix derived for the robotic arm shown in (11), we have the EE’s positional coordinates as

(17) $$P_{x,current}=a_{1}\cos\theta_{1}+a_{2}\cos\theta_{1}\cos\theta_{2}+a_{3}\cos\theta_{1}cos\left(\theta_{2}+\theta_{3}\right)$$

(18) $$P_{y,\;curret}=a_{1}\sin\theta_{1}+a_{2}\sin\theta_{1}\cos\theta_{2}+a_{3}\sin\theta_{1}\cos\left(\theta_{2}+\theta_{3}\right)$$

(19) $$P_{z,\;curret}=a_{0}+a_{2}\sin\theta_{2}+a_{3}\sin\left(\theta_{2}+\theta_{3}\right)$$

The targeted EE coordinates are received from the combined output of the NCAS, IPUPS and the RMAS as *P_x, target_*, *P_y, target_*, and *P_z, target_*. Using the targeted EE coordinates, the positional coordinates shown in (17) to (19), and the loss function equation shown in (15), the following relations are derived:

(20) $$\frac{\partial Є}{\partial \theta_{1}}=2\left(\left(P_{x,target}-P_{x,current}\right)\left(-a_{1}\sin\theta_{1}-a_{2}\sin\theta_{1}\cos\theta_{2}-a_{3}\sin\theta_{1}\cos\left(\theta_{2}+\theta_{3}\right)\right)\;\;\;\;\;\;\;\;\;\;+\left(P_{y,target}-P_{y,current}\right)\left(a_{1}\cos\theta_{1}+a_{2}\cos\theta_{1}\cos\theta_{2}+a_{3}\cos\theta_{1}\cos\left(\theta_{2}+\theta_{3}\right)\right)\right)$$

(21) $$\frac{\partial Є}{\partial \theta_{2}}=2\left(\left(P_{x,target}-P_{x,current}\right)\left(-a_{2}\sin\theta_{2}\cos\theta_{1}-a_{3}\cos\theta_{1}\sin\left(\theta_{2}+\theta_{3}\right)\right)\;\;\;\;\;\;\;\;\;\;+\left(P_{y,target}-P_{y,current}\right)\left(-a_{2}\sin\theta_{2}\sin\theta_{1}-a_{3}\sin\theta_{1}\sin\left(\theta_{2}+\theta_{3}\right)\right)\;\;\;\;\;\;\;+\left(P_{z,target}-P_{z,current}\right)\left(a_{2}\cos\theta_{2}+a_{3}\cos\left(\theta_{2}+\theta_{3}\right)\right)\right)$$

(22) $$\frac{\partial Є}{\partial \theta_{3}}=2\left(\left(P_{x,target}-P_{x,current}\right)\left(-a_{3}\cos\theta_{1}\sin\left(\theta_{2}+\theta_{3}\right)\right)+\left(P_{y,target}-P_{y,current}\right)\left(-a_{3}\sin\theta_{1}\sin\left(\theta_{2}+\theta_{3}\right)\right)\;+\left(P_{z,target}-P_{z,current}\right)\left(a_{3}\cos\left(\theta_{2}+\theta_{3}\right)\right)\right)$$

Based on the above relations, and the GD equation shown in (16), the *θ_i_* values are calculated iteratively as

(23) $$\theta_{1,\;new}=\theta_{1,\;old}+2\eta \left(\left(P_{x,target}-P_{x,current}\right)\left(-a_{1}\sin\theta_{1}-a_{2}\sin\theta_{1}\cos\theta_{2}-a_{3}\sin\theta_{1}\cos\left(\theta_{2}+\theta_{3}\right)\right)\;\;\;\;\;\;+\left(P_{y,target}-P_{y,current}\right)\left(a_{1}\cos\theta_{1}+a_{2}\cos\theta_{1}\cos\theta_{2}+a_{3}\cos\theta_{1}\cos\left(\theta_{2}+\theta_{3}\right)\right)\right)$$

(24) $$\theta_{2,\;new}=\theta_{2,\;old}+2\eta \left(\left(P_{x,target}-P_{x,current}\right)\left(-a_{2}\sin\theta_{2}\cos\theta_{1}-a_{3}\cos\theta_{1}\sin\left(\theta_{2}+\theta_{3}\right)\right)\;\;\;\;\;\;+\left(P_{y,target}-P_{y,current}\right)\left(-a_{2}\sin\theta_{2}\sin\theta_{1}-a_{3}\sin\theta_{1}\sin\left(\theta_{2}+\theta_{3}\right)\right)\;\;\;+\left(P_{z,target}-P_{z,current}\right)\left(a_{2}\cos\theta_{2}+a_{3}\cos\left(\theta_{2}+\theta_{3}\right)\right)\right)$$

(25) $$\theta_{3,\;new}=\theta_{3,\;old}+2\eta \left(\left(P_{x,target}-P_{x,current}\right)\left(-a_{3}\cos\theta_{1}\sin\left(\theta_{2}+\theta_{3}\right)\right)\;\;\;\;\;\;+\left(P_{y,target}-P_{y,current}\right)\left(-a_{3}\sin\theta_{1}\sin\left(\theta_{2}+\theta_{3}\right)\right)\;\;\;\;\;+\left(P_{z,target}-P_{z,current}\right)\left(a_{3}\cos\left(\theta_{2}+\theta_{3}\right)\right)\right)$$

Before starting the actuation process, the robotic arm is kept at the home position, with θ_1_ = π/2, θ_2_ = 0, and θ_3_ = 0. MATLAB R2024b and Arduino Due ensemble are implemented to control the actuation of the joint motors from the home position to the target coordinates as per the GD technique described above. MATLAB’s Arduino support package, containing the ‘Servo’ library and ‘readPosition’ and ‘writePosition’ functions, is used in this study for robotic arm manipulation. The learning rate η is taken as 0.001 and kept the same for all three joint motors. Finally, the robot actuation is conducted at an error threshold value of 0.1 cm^2^ for the reach task.

**Figure 12:**
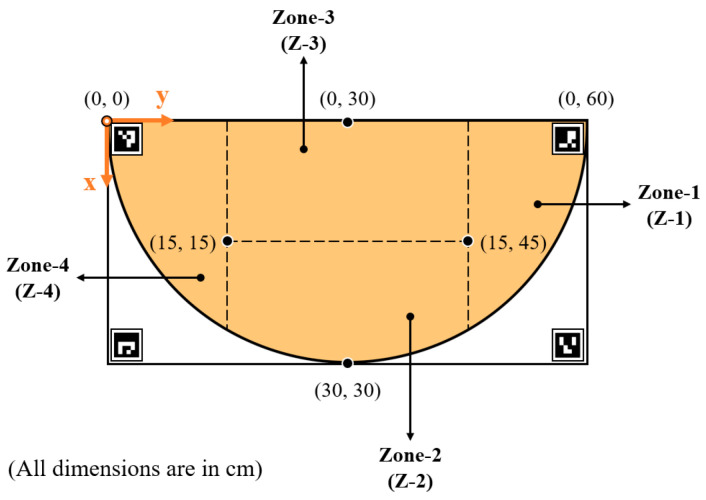
ArUco markers-bounded planar work area and different work zones with 2D positional coordinates for different points defining the work zones.

In the following sections, the above actuation parameters and methodologies are assessed primarily based on the accuracy achieved and the actuation time needed by the robotic manipulator. For this assessment, the study defines a planar work area on the tabletop bounded by the ArUco markers, where the target objects are placed for the NCVAD system. As shown in Figure 12, the defined planar work area is again subdivided into different work zones, viz. Zone-1 (Z-1), Zone-2 (Z-2), Zone-3 (Z-3), and Zone-4 (Z-4). This is designed to facilitate the accuracy and speed assessment of repeated actuations by the robotic arm in various zones of the planar work area. Similarly, the overall latency introduced to the NCVAD system by the GD-based path optimization algorithm based on varying target positions in different work zones is also calculated in this study. Furthermore, in a later section of this study, the defined work zones are also utilized to derive behavioral analysis results for human participants and their performance comparison to the robotic arm in terms of dexterity, handedness, and tactile blind spots.

#### 2.4.3. Accuracy Assessment

In terms of the number of pulses (p), the RDS3115-MG servo motors used for the developed RMAS have a resolution of 50p/revolution. This means that they can only read a minimum actuation value of 0.063 radians. However, the employed GD technique does not have such restrictions and can compute actuation values less than 0.063 radians.

**Figure 13:**
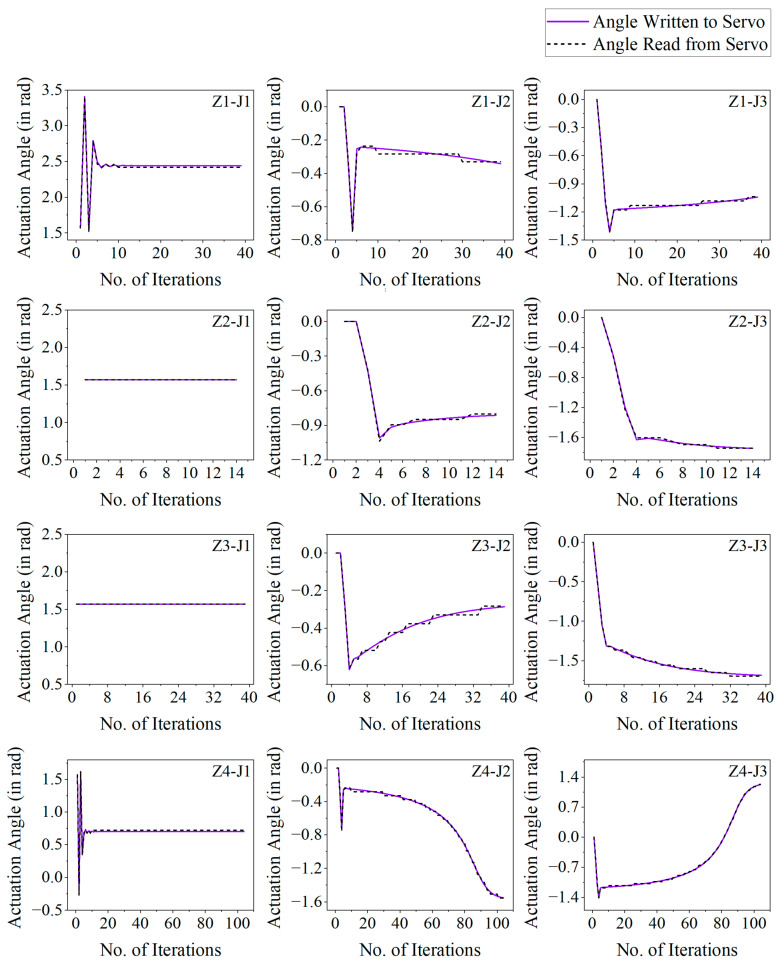
The actuation angle calculated by GD technique (angles written to servo) and the actuation achieved by servo motors (angles read from servo) at joints J1, J2, and J3 plotted for different work zones, Z-1, Z-2, Z-3, and Z-4.

When such a scenario arises, the corresponding motor will not read the actuation values sent to it and, hence, will not change its angular position. To avoid permanent stalling due to this, a dummy variable is introduced in the program, which keeps updating the virtual angular positions as per the GD calculations. When the dummy variable crosses the threshold of 0.063 radians, the motors will be able to read the sent angular position values and actuate accordingly. This results in a stepped curve between the read angular position values from the motors plotted against the number of iterations. At the same time, the plot between the sent angular position values and the number of iterations is relatively smoother. The above discussions are evident from the plots shown in Figure 13, where the desired and actual joint actuations are plotted against the corresponding number of iterations (during the GD optimization process) when the robotic arm is actuated to reach a target placed on different points in the four different work zones (shown in Figure 12).

To determine the accuracy achieved by the RMAS, the intermediate *θ_i_* values derived from the GD technique are written to the respective joint actuators Ji. Similarly, the actual angular position of each joint actuator is read and stored (using the motor’s internal potentiometer readings) against its respective GD iteration. The iterative GD process continues until the positional error of the EE reaches below the defined error threshold value. Therefore, the total positional error of the RMAS is derived by

(26) $$Total\;Positional\;Error=\sqrt{error\;threshold}+f_{FK}\left(\left|Angle\;Written-Angle\;Read\right|\right)$$

Here, *f_FK_* symbolizes the application of forward kinematic functions (defined in (6) through (19) in the previous section) to the difference between the written (desired) and read (actual) actuation angles to convert the angular error at each joint to the positional error at the EE.

#### 2.4.4. Actuation Time to Different Points in the Work Zones

The time needed by the RMAS to reach different work zones in the robotic work area is also determined in this study. For this experiment, the timer starts when the actuation command is sent to the robotic arm, and the time until it reaches the targeted coordinates is calculated. Several points in the four work zones are considered for this experiment, and the average time taken by the robotic arm to perform 30 repeated actuations is determined from this study. Table 3 summarizes the actuation time of the robotic arm for the four zones in the defined planar work area.

#### 2.4.5. Latency Introduced by the Path Optimization Algorithm

To understand the total latency introduced by the RMAS, it is also necessary to understand the time taken for the path planning calculations using the GD-based optimization technique. Similar to the previous experiment, several points in the four work zones are considered for this study, and the average time taken by the robotic arm to perform 30 repeated actuations is determined. For Zone-1, it is found that the average time taken by the optimization algorithm to find a solution is 12.4704 ms for 30 trials, with a standard deviation of 0.6921 ms. Therefore, the maximum time (in the 95% confidence interval) taken by the calculation for a single trial turns out to be 0.4239 ms, as per (5). The average and maximum time needed for different zones are displayed in Table 4.

### 2.5. System Calibration Procedures and Safety Protocols

After discussing the system architecture and the design of various subsystems, it is necessary to follow specific calibration procedures to ensure spatial and temporal coordination among these subsystems.

#### 2.5.1. Camera Calibration and ArUco Marker Placement

The monocular Logitech C270 camera (Robu.in, Maharashtra, India) is calibrated using OpenCV’s ‘calibrateCamera()’ function to facilitate parameter estimation of the camera system. With this, the parametric and coefficient information is obtained, which is necessary to determine an accurate relationship between a three-dimensional point in the real-world and its two-dimensinal counterpart on the image plane. There are typically two types of these parameters: internal parameters (e.g., distortion coefficients, focal length, optical center) and external parameters (referring to the rotation and translation of the camera with respect to the world coordinate system). A standard checkerboard pattern with 7 × 6 grid size (known dimensions of 1 × 1 cm) is used to capture multiple images with the monocular camera from varying angles and distances to derive the intrinsic camera matrix and distortion coefficients. The focal length, principal point, and radial distortion values are registered to ensure accurate pose estimation during coordinate transformation using ArUco markers.

After the camera calibration, four ArUco markers with unique IDs (shown in Figure 4) and printed with fixed dimensions of 50 × 50 mm are used to transform image coordinates to physical coordinates and vice versa. First, the 600 × 300 mm work area is defined by placing the printed markers carefully on the four corners, as shown in Figure 12. Additionally, the ArUco markers are laminated to prevent wear and ensure reliable readings across multiple trials. Afterward, the coordinate frame origin is assigned to the marker with ID ‘0’, and OpenCV’s ‘cv2.aruco.estimatePoseSingleMarkers()’ is used to retrieve the rotation and translation vectors of the base marker (origin with ID ‘0’) with respect to the camera. Then, the Perspective-n-Point (PnP) algorithms are applied to continually use the calibrated camera-cum-ArUco setup for 3D point projection to 2D and transformation from image coordinates to real-world coordinates.

#### 2.5.2. Robotic Arm Calibration and EEG-to-Actuation Synchronization

Since the RMAS depends on the input from other subsystems, such as the target coordinate values derived using the ArUco-based physical position determination, it is important to calibrate the robotic arm and ensure its home position is in sync with the calibrated camera-cum-ArUco setup described earlier. Home positions of all three servo joints are defined as θ_1_ = π/2, θ_2_ = 0, and θ_3_ = 0 radians. The actual servo positions are also verified using the ‘readPosition()’ command to access the feedback from the RDS3115-MG motors’ internal potentiometers. Finally, when at the home position, a basic portable laser pointer is used to physically verify that the servo 1 representing joint 1 (J1 shown in Figure 10a) of the robotic arm is vertically above the coordinate (0 cm, 30 cm) of the work area shown in Figure 12. Likewise, the laser pointer is used to also verify that the base of the robotic arm is placed exactly parallel to the line connecting markers with ID ‘0’ and ‘1’. For all the above calibrations and validations, the center of the base marker (ID ‘0’) is taken as the origin of the coordinate frame attached to the table top. Furthermore, in the above process, the error margins are kept within a tolerance of ±2 mm, and dry runs are conducted before each trial to ensure the positional calibrations are maintained by the robotic arm.

Furthermore, the synchronization between the EEG-based selection and robotic arm actuation is achieved using the ‘time’ library on the Python environment. With this timestamp-based signaling in the Python environment, the audio prompt and the corresponding EEG classification event are registered using the ‘time.time()’ function. Individual subsystem latency studies conducted in previous Section 2.3.5 and Section 2.4.5 using the same method suggest that a maximum time of 0.4532 ms is spent between the start of jaw clenching and the end of the GD-based path optimization calculations. Compared to a normal human’s reaction time [60,61,62], the observed time difference of < 1 ms is considered suitable for real-time interaction between the subsystems. In conjunction with the aforementioned dry run, a drift check is also executed for the overall system every ten trials, where any deviation (greater than ±2 mm or 1 ms) from the baseline is compensated by repeating the above calibration methods. Following these calibration, verification, and drift compensation steps, the spatial and temporal alignment between different subsystems and their components is ensured in the developed NCVAD system.

#### 2.5.3. Safety Protocols for Error Handling

Given the intended use of the NCVAD system by visually impaired individuals, robust and user-safe operations need to be ensured in the system development. Therefore, different error handling protocols are maintained for the following scenarios:

1. EEG data misclassification.
2. ArUco marker occlusion and visual obstruction.
3. Servo actuation mismatch.
4. Emergency override.

The developed SCDC algorithm is tuned to prioritize the successful classification of jaw-clenching activities (label ‘1’) to ensure higher recall value and facilitate EEG misclassification handling. This already enables the reduction in false negatives ensuring preventive error handling, as described in the earlier sections. In addition to this, a future scope of the project may include a real-time prediction loop with multiple classification windows, where the NCAS registers a signal as label ‘1’ only if three or more consecutive classification results consistently output the same label. Using this, the false positive rates can be reduced along with the false negatives with the expense of added latency.

To address the visual obstruction issue concerning ArUco markers, the current IPUPS registers an unsuccessful detection only if fewer than three markers are visible or if the base marker (ID ‘0’) is not detected. Marker lamination is adopted as a preventive measure for this issue. Likewise, the dry run and drift check trials are executed periodically as mentioned in Section 2.5.2 to avoid running into issues similar to marker occlusion. Additionally, if for some reason, marker occlusions occur during the experiment, then the system is programmed to display an error message stating “ArUco not found; please recalibrate.” When such a situation arises, the root cause of the problem is identified and resolved, and all the calibration processes are reiterated to restart the experiment.

Similarly, to ensure that the servo motors are actuating within the defined work area, the current position of each servo is read during each actuation loop. Using this, the RMAS is programmed to continuously monitor the actuation angles, and stop the servos at place whenever the error between the desired and actual servo angular positions exceeds beyond 0.1 radians. This triggers the “servo fault” error message, which is then resolved following the same root cause analysis and recalibration methods as mentioned above.

Finally, it is acknowledged that some unforeseen situations may arise during the experiment. While primary error-handling measures have been incorporated into the system design, in the event of unpredictable system behavior, the user/operator should be able to quickly stop the moving robotic arm using an emergency stop mechanism. This override is implemented using a parallel thread to react to any key press on the laptop keyboard. Using the keyboard.read_event() function in Python, the fail-safe mechanism is programmed to ensure that any key pressed during an on-going experiment acts as the emergency override. Additionally, in case of power line failures, a physical kill switch is also connected to the power supply unit, enabling quick disconnection and ensuring the safety of the user and system components.

## 3. Results of the Experimental Validation of the Developed Physical NCVAD System as a Whole

After a detailed description of the system architecture and the design and performance parameters of different subsystems in the previous sections, this section is dedicated to real-time validation of the combined performance of all the subsystems using a developed physical model of the NCVAD system as a whole.

### 3.1. Experiment Design for Physical System Validation

For this experiment, the EEG signal was acquired from one male volunteer, 28 years of age. The neural data acquisition experiment was conducted at the VRRL Laboratory at the Indian Institute of Technology Madras, Chennai, India. The experimental procedure adheres to the Declaration of Helsinki and follows all the necessary ethical guidelines approved by the ethics committee of the Indian Institute of Technology Madras, with the proposal number IEC/2024-01/PVM/19, as described earlier. A detailed description of the experimental procedures was provided to the volunteer, and his consent was obtained prior to the start of the experiment.

The volunteer was asked to sit comfortably in a chair wearing the Emotiv EPOC Flex EEG cap. Four ArUco markers (shown in Figure 4) were placed on a horizontal table top to create the planar work area described in Figure 12. A Logitech C270 monocular camera was fixed at a set height and angle to enable a clear view of the objects placed inside the planar work area at all times. The 3D-printed 3-DoF robotic arm was fixed on a platform on the table top to satisfy the work area alignments and dimensions shown in Figure 11 and Figure 12. The three RDS3115-MG servo motors used to design the joints of the robotic arm were actuated by the control algorithm running on an i7-13650HX Windows laptop with a connection to the Arduino Due circuit and a high-accuracy Agilent E3634A DC power supply unit. This unit was set to supply 7 V voltage with a 1.0 A cutoff for over-current protection. Furthermore, the same laptop was responsible for sequentially deploying all the necessary software and algorithms (YOLOv8n-based object detection, audio prompting, EEG signal processing, SCDC classification, and robotic arm manipulation) as per the schema of information transfer shown in Figure 1.

**Figure 14:**
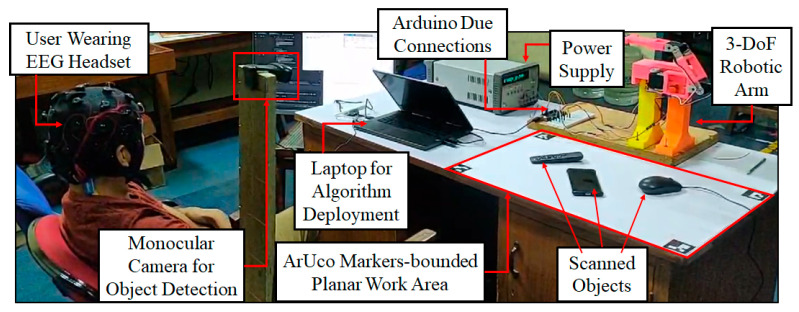
Developed physical experiment setup depicting different components of the NCVAD system as a whole.

Once the experiment began, the volunteer was instructed to clench his jaws to initiate the object detection algorithm whenever he felt ready to do so. Once the volunteer’s jaw-clenching action was detected from the EEG signal, the EPS uses the captured image frame to detect the object(s) kept inside the work area defined above. After this, the names of the detected object(s) are relayed to the volunteer as an audio prompt. This helps the volunteer get an idea of what objects are placed in front of him and select the object he wants to reach. Afterward, the system repeats the names of the same detected objects in the same sequence one by one with a gap of 1500 ms between each dictation. This allows the volunteer to clench his jaws again when the audio prompt dictates the name of his intended object. Once this is completed, the SCDC algorithm registers a jaw clench (with classification label ‘1’) by analyzing the volunteer’s neural signal streamed continuously to the laptop which controls the algorithm deployment. Then, as per the detected target’s positional coordinates, the GD-based path optimization algorithm actuates the robotic manipulator to reach the object selected by the volunteer. Figure 14 depicts the experiment setup and its constituent components for the developed physical model of the NCVAD system.

### 3.2. Validation of Results and Comparison with Similar Work in the Field of Study

The above experiment was run 30 times to derive key performance parameters of the NCVAD system. Out of the 30 experiments, the system correctly detects jaw clenching 26 times (true positives). This results in a net accuracy of 86.67%. Compared to this work, Velez et al. [27] achieved 18 correct jaw-clenching detections out of 20 test detections using a Neurosky Mindwave Headset. Similarly, in another work by Masud et al. [63], an average accuracy of 87.5% was achieved by utilizing a P300 event-related potential-based paradigm to classify neural data for numeric/symbolic option selection to assist persons with paraplegia. Therefore, the developed NCVAD system demonstrates performance that is on par with established work in the field of neural signal classification and assistive technology.

In addition, one false negative was noticed in the above 30 trials conducted for the NCVAD validation experiment, which means that the system detected no clenching (classification label ‘0’) even though the volunteer performed the jaw-clenching action (classification label ‘1’). Similarly, there were three false positives, where the system detected jaw clenching (label ‘1’) when no jaw clenching was performed by the volunteer (label ‘0’). Using the formulae shown in (2) and (3), the precision and recall (also called sensitivity) values for this validation experiment are calculated to be 89.65% and 96.29%, respectively. Since the neural data is received as a data stream and decoded continuously at 8 SPS, the specificity of the developed system is well above 90% as among multiple true negatives (label ‘0’) only three false positives are detected. Compared to the achieved results, Khoshnam et al. [28] proposed a method for online jaw clench detection with a sensitivity of 80% and a specificity of 88%. Therefore, the developed NCVAD system is able to outperform similar classification models introduced by previously published work on jaw-clenching detection.

Furthermore, the observed values of precision and recall also satisfy the results obtained during the training and validation of the SCDC algorithm, as explained in Section 2.3.4. As mentioned earlier, to meet the primary goal of the SCDC algorithm, misclassifying a label ‘1’ proves more expensive than misclassifying a label ‘0’. While not always true, there is often an inverse correlation between recall and precision, as the number of false positives and false negatives are inversely related to each other for a given accuracy achieved by the classifier model. Therefore, the SCDC algorithm is tuned to achieve recall values higher than its corresponding precision value by reducing the number of false negatives. This argument substantiates the above values of precision and recall obtained from the current validation experiment. In conclusion, the SCDC model, implemented in the physical NCVAD setup, successfully validates the previous algorithmic experimental results and achieves performance on par with or exceeding previously established studies in the field of neuronal assistive technology.

Similar to the classification accuracy achieved by the NCVAD system as a whole, the total time taken by the system to successfully guide the robotic arm to the target coordinates is also assessed as a part of this validation study. During the 30 trials, the system takes an average of 610.25 ms to complete the object detection, text-to-speech conversion, jaw-clenching classification, and robotic arm actuation tasks. The time taken for these tasks is obtained by subtracting the dictation time (with a 1500 ms delay between consecutive sentences) from the total time taken by the NCVAD system. This is because the dictation time varies based on whether the system mentions the target object first or toward the end. If it is dictated towards the end, the volunteer has to wait longer before hearing the name of the target and performing jaw clenching to select it. Therefore, the maximum time for this experiment is considered for the speed assessment of the NCVAD system, which adds up to an average of 26.3 s over 30 trials. Table 5 shows the experimentally validated time taken by different subsystems on average, adding up to the overall time taken by the NCVAD system as a whole.

Compared to the above latency achieved by the current NCVAD system, the P300 event-related potential (ERP)-based paradigm, implemented by Akram et al. [64], took a total of 31.5 s for the selection process. While the P300 ERP has its advantages in neural signal processing, it requires a list of multiple options to distinguish the target from the rest in the list. This might make such models underperform when a small number of options are presented with an equal chance of being selected. Therefore, the developed NCVAD system, with jaw clenching as the trigger, may prove faster in such scenarios involving target selection, as substantiated by the results obtained from the above experiment. Table 6 summarizes the above discussion on relative performance of the present methodologies compared to previous studies and benchmarks.

In addition to the above performance comparison with cotemporary research, it is important to note that relying on processes involving ERPs and SSVEPs may prove counter-intuitive when developing systems for visual assistance, as such approaches often depend on vision-based triggers. Similar concerns may arise during the implementation of jaw-clench based BCI development, where users might not be able to perform jaw movement. To cater to these real-world adoptability and usability concerns, the present NCVAD system has been designed using a modular architecture. This allows the core concepts demonstrated in the above experimental setup to be extended using other technologies in the field, without deviating from the present scope of the project.

For example, let us consider an example where the EPS incorporates a wall-mounted camera with enhanced field of view, and the RMAS guides a wheeled robot instead of a fixed robotic arm. This enables the RMAS to reach any target within the EPS’s field of view, as in the original setup, but with improved scalability. In such a scenario, the robot’s trajectory can be dynamically adjusted to reach different target coordinates from its home position, whenever the user clenches their jaw to make a selection. Therefore, the modular system design of the NCVAD allows it to cater to real-world usability, portability, and scalability concerns beyond the confines of a laboratory environment. These potential enhancements can be explored in future design iterations by using the results of the present preliminary analysis as a foundation for further development.

## 4. Latency Results on Human Performance Evaluation

Establishing a fully functional human-in-the-loop assistive system necessitates a clear understanding of different temporal parameters of human performance, primarily regarding human response and reaction time. The current study has designed two experiments to assess the temporal properties pertinent to human interaction with the NCVAD system. One experiment aims to record the time needed by a user to generate a selection command after receiving the audio prompt for choosing the targeted object, and the time for the Python environment to register the generated command. The other experiment is designed to determine different statistics corresponding to the task execution time of different people to reach and identify the targeted object placed among decoys in a set work area. This study aims to derive key insights regarding user behavior from the above experiments, which, in turn, will enhance the expected utility of the developed NCVAD system.

### 4.1. Experiment-1: Selection Command Generation and Registration

#### 4.1.1. Experiment Design for Experiment-1

Experiment-1 of the human performance evaluation trials is designed to replicate the audio prompting segment of the IPUPS manually. This aims to assess the time difference between the delivery of an audio prompt and the participant’s jaw-clenching response, which is used to register their selection. The primary steps of this replication involve a ‘selection statement dictation’ (SSD) step by the system and a ‘selection command generation’ (SCG) step by the participant. In the SSD step, a varying set of audio messages (statements), which contain information about virtually created objects and are structured similar to an actual IPUPS prompt, is presented to the participants over ten distinct trials. While designing the experiment, it is kept in mind that in a real-life scenario, where a visually challenged person is using the NCVAD, the objects and the order in which they appear to the EPS are nondeterministic. Hence, the IPUPS does not follow any specific patterns while dictating the names of the detected objects to the user. Therefore, during the experiment, the number of virtual objects in the SSD is varied randomly between five and eight, and their respective names are also jumbled in each trial to induce randomness in the experiment. This adds to the validity of the aforesaid replication of the actual prompting process in a real-life scenario.

Then, in the SCG step, the participant has to select the predefined target upon hearing the corresponding ‘target statement’ during the SSD. The selection command by the participant is recorded by the ‘keyboard.read_event()’ function in the Python environment, and the time required for registering this selection command from the start of the ‘target statement’ dictation is recorded as the reaction time of the participant with the ‘time.time()’ function. The multi-threading capabilities of the ‘pyttsx3’ library are utilized in this experiment to record the reaction time corresponding to the SCG in parallel with the SSD, without any loss of relevant information.

To provide further clarity on the experiment design, the following example is considered with five objects, where one object is the target and the other four are decoys. Here, the participant hears the following statements during the SSD step:

*“The number of objects detected are five. They are mouse, cellphone, remote, book, pen. Please select any.”*

*“One is mouse.”*

*“Two is cellphone.”*

*“Three is remote.”*

*“Four is book.”*

*“Five is pen.”*

Similar to the original IPUPS, the statements are read at 200 WPM, and there is a 1500 ms delay between the end of each statement and the beginning of the next. This allows the participants to easily comprehend the object names and register their selection command for the target. In the above example, with ‘cellphone’ as the targeted object, the participant registers their selection after the fifth statement during the SCG step, and the reaction time is noted in the Python environment.

#### 4.1.2. Participants and Experiment Results for Experiment-1

**Figure 15:**
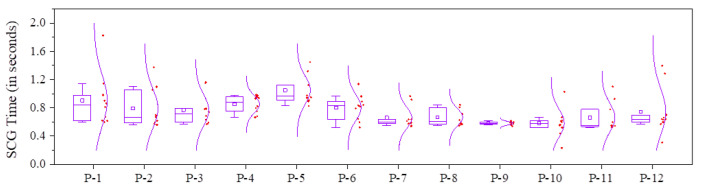
Box-and-whiskers plot and the individual datapoints fitted to a normal distribution curve displaying the variation in selection command generation time achieved by different participants (P-1 to P-12) during experiment-1.

The experiment was conducted at the VRRL Laboratory at the Indian Institute of Technology Madras, Chennai, India. Twelve healthy participants (nine males and three females) with an average age of 26.67 (SD: 3.6) volunteered for this experiment. Informed consent was obtained from all the participants before the start of the experiment, and it was ensured that the experiment adhered to all the pertinent ethical guidelines as per the ethics approval details provided in the earlier section. In this experiment, all the participants achieved a mean average SCG time of (0.756 ± 0.14) seconds. Figure 15 demonstrates the variations in SCG time achieved by different participants over ten different trials, where the twelve participants are represented by symbols P-1 to P-12. In the figure, the box-and-whiskers plot as well as the original datapoints with a fitted normal distribution curve are shown for each of the twelve participants.

#### 4.1.3. Behavioral Analysis for Experiment-1

Based on the participants’ feedback and results, several key behavioral points are observed for the variations in different participants’ SCG time. In some trials during the experiment, participants waited for the ‘target statement’ to conclude and registered their SCG after ensuring the validity of the statement. However, after an average of three iterations, some participants began to use the first and second statements of the SSD to take hints about the position of the ‘target statement’. Considering the previous example, with ‘cellphone’ as the target object, some participants can deduce that the fifth statement is going to be: “Two is cellphone.” Therefore, they try to ‘pre-register’ their selection command even without explicitly hearing the statement. In other words, they register their selection early by ‘timing the target statement’ even before the sentence finishes. This states that upon extended and multiple use of the NCVAD system, it is possible for a user to become accustomed to the system, which improves the utility of the human-in-the-loop device.

The aforementioned ‘pre-registration’ results in very low reaction time for some participants (P-10 and P-12). However, it is also true that the participants could not always correctly ‘time the target statement’. Due to this, they had to miss at their first try and then immediately reattempt to register their selection command again, all within the stipulated time of the aforementioned 1500 ms duration. This results in outliers in their SCG time, as shown in Figure 15. However, it has to be noted that the average speed of these participants is still higher than others who are usually slow or do not tend to ‘time the target statement’ (P-1 and P-5). Some participants also displayed abrupt behaviors where the SCG is delayed without any external influences, resulting in outliers (P-7). As per the participant feedback after the experiment, the most likely reason is the participant relaxing on a few iterations while trying to beat their best speed on other trials. The above argument is evident from a low mean and inter-quartile range (IQR) for P-7 with two outliers, as shown in Figure 15. The above figure also establishes that all the participants’ SCGs are always within a minimum and maximum value, and the participants are able to register their selection commands within the 1500 ms time window in 100% of the trials. The above findings are intended to prove useful in optimizing future variations in the NCVAD system.

### 4.2. Experiment-2: Blindfolded Reach Task with Target and Decoys

#### 4.2.1. Experiment Design for Experiment-2

The developed RMAS actuates a robotic arm to reach targeted objects placed in a defined planar work area, as described in Figure 12. Experiment-2 is designed to assess the time taken by the NCVAD system to complete the reach task in comparison to humans. Here, participants are asked to perform the reach task in a work area similar to the RMAS robotic arm to derive comparative results on the human and robotic performance on equal ground.

As discussed earlier, the robotic arm of the RMAS is designed with dimensions that are approximately half the anthropometric values for an adult human. Therefore, in this experiment, all the objects are placed in an area that is a replica of the robotic arm’s planar work area, while the dimensions are multiplied by a factor of two. The work zones are similar and proportional to the four zones created for the robotic arm experiment conducted to assess the RMAS actuation speed. In the current experiment, one target and two decoys are placed in front of a blindfolded participant in the set work area. The experiment records the time taken by the participant to reach the four different work zones and identify the predefined target placed in one of them. The participant is free to move their arm in any manner to ‘scan’ the area and find the target; however, they can use only one arm positioned at the center of the semicircular work area. This is performed to facilitate the comparison of the human response time to the actuation speed of the RMAS with a single manipulator. The target is kept the same for all the multiple trials of individual participants in order to simulate the heightened experience of blind users with recognizing daily objects from touch percepts. Twenty trials are conducted per participant by randomly placing the target five times each in one of the four zones.

#### 4.2.2. Participants and Experiment Results for Experiment-2

**Figure 16:**
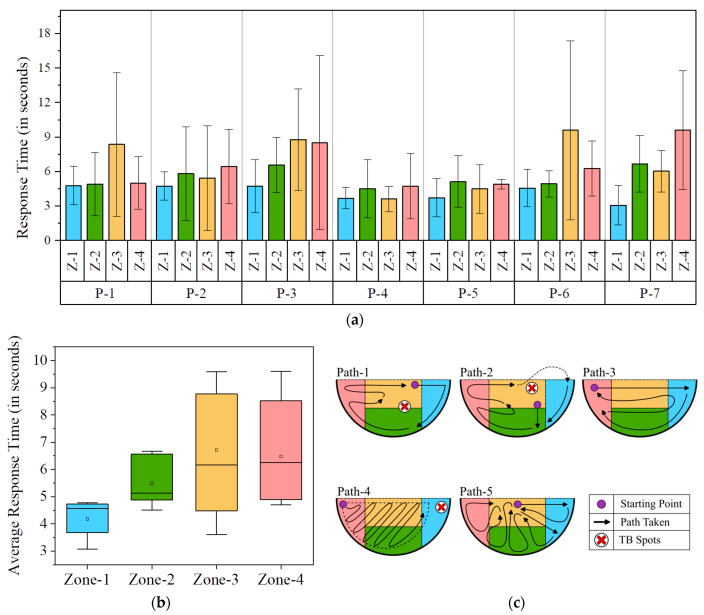
(**a**) Variation in response time achieved by the blindfolded participants (P-1 to P-7) to reach target placed in different work zones. (**b**) Comparative average response time achieved by all participants in different work zones. (**c**) Typical hand-tracing paths followed by different participants during experiment-2.

The experiment was conducted at the VRRL Laboratory at the Indian Institute of Technology Madras, Chennai, India. Seven healthy participants (six males and one female) with an average age of 27.29 (SD: 4.4) volunteered for this experiment. Informed consent was obtained from all the participants before the start of the experiment, and it was ensured that the experiment adhered to all the pertinent ethical guidelines as per the ethics approval details provided in the earlier section. All the participants were right-handed and conducted the experiment with their right hands, while being blindfolded. Figure 16 demonstrates different statistics varying with different participants and work zones during the reach experiment, where seven participants are represented by symbols P-1 to P-7, and four work zones are represented by symbols Z-1 to Z-4.

#### 4.2.3. Behavioral Analysis for Experiment-1

Five typical hand-tracing paths followed by the participants are shown in Figure 16c. These followed paths influence the statistics obtained for different participants’ response times in different zones. One such influence is the creation of ‘tactile blind spots’ (TBS). For example, participants following path-1 often neglected the objects placed in the top left half of Z-3 (marked by ‘X’), and hence, took a larger number of scans along their hand-tracing path, before finding the target, if placed in that spot. Similarly, participants following path-2 had a TBS created on the bottom left half of Z-3. This results in a longer response time by participants to find the target when placed on their respective TBS. Further, participants reached the bottom right half of Z-3 relatively late, irrespective of whether they chose path-1 or path-2. This results in a high variance in the response time achieved by different participants for Z-3 (P-1, P-2, P-3, P-6), as evident from Figure 16a.

In addition to that, even though the work area is symmetric with respect to the participants’ hand positions, their right-handedness forms a natural TBS towards the left corner of zone-4 due to their usual clockwise hand-tracing method. This results in a high amount of time being taken to find the target placed on the said spot and a relatively short time when the target is placed elsewhere in the same Z-4. This condition is also valid for P-7, who followed path-4 during the experiment, resulting in a TBS in Z-4. This argument is substantiated by the large IQR for Z-4, as shown in Figure 16b, as well as the high standard deviation for Z-4 achieved by the response times of P-7 (Figure 16a). While the right-handedness and clockwise hand tracing deteriorate some parts of the performance in Z-4, they positively add to the performance in Z-1, which is placed towards the right half of the work area. The participants reported higher control over the right half of the work area, and this statement is validated by the short time taken to find the target placed in Z-1. This results in low mean and IQR values for Z-1 when the combined performance of all participants is considered, as shown in Figure 16b.

In contrast, when path-3 is followed as the hand-tracing path, it takes advantage of the clockwise motion and the right-handedness of the participants and results in the least TBS. Due to the lack of TBS, path-3 results in the best coefficient of variation (CoV), demonstrating a lower mean and lower variations, as obtained by P-5. While P-5 obtains a CoV of 0.352, P-4 achieves a CoV of 0.446 despite having a 9.42% lower mean response time averaged over all zones. While the path followed by P-4 (path-1) is similar to path-3 to some extent, the presence of TBS affects the overall performance, which is captured in the multiple trials of this experiment. This tells us about the adverse effects of TBS on the response time of different participants and the importance of optimizing the hand-tracing path for maximum performance.

## 5. Discussions on Human Performance in Comparison to the Robotic Arm Actuation

Even with optimized hand tracing, the human response falls behind a vision-powered automated robotic system, such as the NCVAD, in some aspects. To take an example from the conducted experiments, until the participant actually found the target, most of their effort was wasted in ‘rummaging’ around the work area. Such wasted effort often adds to the frustration of a visually impaired person, apart from adding physical exhaustion. Additionally, the situation degrades drastically if the user is present in a new or dynamically changing environment, such as an office space or factory floor, making such environments excessively inaccessible and unsafe for the person.

Furthermore, by rummaging around in the set work area, the human response time varies from person to person and from one zone of the work area to another, resulting in a high variance and a high average CoV value of 47.57%. However, the results are significantly improved with highly stable actuation timings when a robotic system conducts the same reach task (average CoV 8.28%), as evident from the robot arm actuation experiment in the previous section. This is primarily due to the absence of TBS for the NCVAD system. While optimizations are possible in terms of efficiency, the EPS will always guide the RMAS to reach the target placed anywhere in the planar work area bounded by the ArUco markers.

One more observation from the above experiment is that in order to achieve high response speeds, participants often moved their arms randomly and aimlessly at high speeds, knocking objects over, and sometimes even pushing the target out of the work area. In real-world scenarios, when the objects are placed on an elevated surface, the target might fall off the surface, adding to the woes of a visually challenged person. Such scenarios force many visually challenged individuals to rely on others for simple tasks like reach and grasp of daily objects. Contrarily, a vision-powered robotic arm can easily be programmed to avoid bumping into potential targets. In the current NCVAD system, the end effector of the RMAS is placed at a height higher than the maximum height of the potential targets, and it lowers at the end of the actuation after being vertically above the target. This is achieved by actuating J-2 preceded by J-3. Furthermore, the implementation of robotic assistance becomes necessary for the safety of a visually challenged person in the presence of sharp objects that might hurt the person if approached without caution. The NCVAD system can be programmed to alert the user when the EPS detects a potentially harmful object. Additionally, the EPS and RMAS can be integrated to approach the sharp object from a safe side (its handle) when the user selects to reach the target object. Such features can be further optimized to resolve and circumvent common problems that can add enormously to the quality of life of visually challenged individuals.

## 6. Usability Standard and Benchmarks

To provide a rigorous framework for our usability evaluation, the ISO 9241-210:2019 and ISO 9241-11:2018 standards on “Ergonomics of human–system interaction” are adopted for benchmarking [65,66]. In accordance with the ISO 9241-11 standard, the user evaluation in the current study is designed to capture key metrics as shown in Table 7.

The effectiveness and efficiency metrics are represented by the task accuracy and task completion times, respectively, described during the physical system evaluation in Section 3. This section also compares the obtained accuracy and speed of the current NCVAD system to peer-reviewed frameworks in the field of study. Additionally, the achieved accuracy of 86.67% is found to be within the typical range of 75–90% reported in the passive BCI integration frameworks proposed by Zander & Kothe [67]. Similar to passive BCI frameworks, EEG-informed usability studies by Frey et al. [68] highlight continuous cognitive load monitoring during interface tasks, demonstrating the correlation of neurophysiological strain with efficiency and satisfaction. Though the present study does not explicitly provide deeper analysis on neural workload, the temporal variability analysis of user actions and the NASA-TLX score assessment included at the end of this section is expected to provide key insights into the overall perceived workload of the developed system.

The cognitive and physical load on the participant during the experimentation process is assessed using a Hart and Staveland’s NASA Task Load Index (TLX) method using a 21 graded seven-point scale [69]. Table 8 summarizes the participant’s responses obtaining an average scaled score of 25 across subscales. This suggests that the developed NCVAD system demonstrates strong user-friendliness with low overall workload.

Furthermore, to evaluate the user satisfaction metric, the System Usability Scale (SUS) [70] is calculated through post-session questionnaires shown in Table 9. A five-point Likert scale (from “Strongly Disagree” to “Strongly Agree”) is used to assess the participant’s subjective view of the NCVAD’s usability after the trial on the physical setup is concluded. The evaluation procedures of the SUS involve assigning scores to odd-numbered items as (response—1) and even-numbered items as (5—response), and multiplying 2.5 to the total score obtained [71,72,73]. Following the evaluation procedure mentioned above the current system achieves a strong usability score of 82.5.

The preliminary feedback from the participants is recorded for future implementations and improvements. In summary, the primary positive feedback involved design simplicity of the well-integrated subsystems, which helped the participant to understand different functionalities quickly. Additionally, the user reported that continuously wearing the EEG headset for the duration of the experiment was fine. However, it might not be acceptable by most people to keep wearing the headset throughout the day. To resolve this, a lighter design of the EEG headset is under development with optimized electrode numbers and placement. Future large-scale user studies on the improved versions of the NCVAD are expected to provide more insights into the system’s usability and corresponding user satisfaction levels.

## 7. Conclusions and Future Scope

This work successfully establishes the feasibility of the fusion of BCI and object detection algorithms toward actuating a neuronally controlled robotic device to assist visually challenged individuals, using simple jaw-clenching triggers. The environmental perception subsystem powered by YOLOv8n is capable of recognizing the targeted objects with a tested mAp of 37.3 and an inference speed of 80.4 ms. Similarly, the maximum precision and recall values achieved by the neural command acquisition subsystem is more than 85% during the EEG data classification process, which takes less than 37 µS to decode each datapoint. Likewise, the Robotic Manipulator Actuation Subsystem can reach the positional coordinates of the user-selected object with an average SSE value of 0.1 cm^2^ while taking an average maximum time of 0.5484 s for the GD-based path optimization and robotic arm actuation. Combinedly, the NCAVD system as a whole achieves an average end-to-end latency of 26.3 s, including the user prompting activity by the IPUPS. The above results are also validated by the experimental assessment of a developed physical model of the NCVAD system. Compared to the response time for the reach task by humans, the robotic arm (RMAS) demonstrates an increase of 773.49% in reach time and an improvement of 474.47% in the CoV value, averaged over different work zones. Furthermore, the study conducted to determine the usability of the developed system results in an SUS score of 82.5 and a scaled NASA-TLX score of 25, suggesting strong user-friendliness with low perceived work load achieved by the system. In summary, this study establishes that by enabling selection through EEG neural signals, the developed NCVAD gives a sense of natural and active control to its user even while using robotic assistance.

The current text-to-speech (TTS) system is designed to add fixed delays between each dictated statement during the auditory prompt delivery process. However, as observed during this study, some users can naturally reduce their selection command generation time during the target statement dictation process. The future scope of this system may incorporate dynamically adjusted dictation, which caters to the personalized reaction time of individual users. Furthermore, the current study employs healthy volunteers to establish and demonstrate a preliminary proof-of-concept of the proposed neuronally controlled visual assistive device. Future research will focus on refining neural signal processing and hardware complexity to extract relevant information from neural signals reliably by deploying more sophisticated machine/deep learning algorithms, and easy-to-use EEG headset with optimized number of electrodes. In addition, a future iteration of the NCVAD design will be validated through large-scale user studies on profoundly blind volunteers to optimize the system’s efficacy in daily life scenarios. Contrary to the present single-subject validation, the large-scale study is expected to provide better generalization of the SCDC model’s classification accuracy and the NCVAD system’s physical utility. Such improvements will significantly bolster the system’s adaptability and validate its targeted applications, including advanced neuroprosthetic assistance, rehabilitation systems, and industrial human–robot interaction [16].

## Figures and Tables

**Table 1:** Technical specifications of hardware components.

Hardware Component	Respective Activity	Parent Subsystem	Technical Specification
Monocular Camera	Image Capturing	EPS	Make: Logitech C270 HD Resolution: 720p at 30 FPS Sensor: 0.9 MP CMOS Field of View: 55–60° Interface: USB-A 2.0 Dimensions: 72.9 × 31.9 × 66.6 mm Compatibility: Windows 7+, USB-A Type: Monocular Vision System
Speaker System	Audio Prompting	IPUPS	Make: Realtek ALC3287 Power Rating: 2 W Type: Stereo Sound System
EEG Headset	Neural Data Acquisition	NCAS	Make: Emotiv EPOC Flex EEG Saline Kit No. of Channels: 32 Referencing: CMS/DRL Configuration: 10–20 international system of placement Resolution: 16-bit ADC Filter: Digital fifth order sinc filter Notching: 50 Hz Bandwidth: 0.2–45 Hz Sampling Rate: 2048 Hz (internal); 128 SPS (EEG stream); 8 SPS (FBP stream) Connectivity: 2.4 GHz USB receiver
Servo Motors	Joint Actuations of the Robotic Arm	RMAS	Name: RDS3115-MG Current: Direct Current (DC) Rotation Angle: 0–270° Operating Voltage: 4.8–7.2 V Torque Rating: 13.5–17 kg-cm Current Drawn: 4–5 mA (idle); 1.8–2.2 A (stall) Dimensions: 40 × 20 × 40 mm
Power Supply Unit	Power the Servo Motors for Joint Actuation	RMAS	Make: Agilent E3634A DC Output Range: 0–25 V, up to 7 A Power Rating: 175–200 W Voltage Accuracy: ±(0.05% + 10 mV) Current Accuracy: ±(0.2% + 10 mA) Noise: <500 µV_rms_/3 mV_p-p_; <2 mA_rms_ Interfaces: GPIB; RS-232 Protection: Over Current (OC) and Over voltage (OV) protection Form Factor: 213 × 133 × 348 mm; 9.5 kg
Embedded Controller	Send Control Signal to the Servo Motors	RMAS	Make: Arduino Due Microcontroller: Atmel SAM3 × 8E ARM Cortex-M3 Microcontroller Spec: 32-bit; 84 MHz Flash Memory: 512 KB SRAM: 96 KB (64 + 32 KB) Operating Voltage: 3.3 V Digital I/O Pins: 54 Serial Ports: 4 × UART Total I/O Output Current: 130 mA Board Size: 101.5 × 53.3 mm
Laptop	Algorithms Deployment and Overall Task Control	All Subsystems	OS: Microsoft Windows 11 Version: 10.0.26100 System Type: x64-based PC Processor: i7-13650HX, 2600 Mhz Cores: 14 RAM: 24.0 GB Interface: Intel(R) USB 3.20

**Table 2:** DH parameters for the 3-DoF NCAS robotic manipulator.

Joint	*θ*	*α*	*a*	*d*
J1	θ_1_ *	π/2	a_1_	a_0_
J2	θ_2_ *	0	a_2_	0
J3	θ_3_ *	0	a_3_	0

* Symbolizes a joint variable as per DH parameter conventions.

**Table 3:** Actuation time taken by the robotic arm to reach different points in the four work zones.

	Average Time Taken (in Seconds)	Standard Deviation (in Seconds)	Maximum Time Needed with 95% CI (in Seconds)
Zone-1	0.556	0.071274	0.600176
Zone-2	0.414	0.036469	0.436604
Zone-3	0.480	0.053385	0.513089
Zone-4	0.620	0.035355	0.641913

**Table 4:** Calculation time taken by the GD optimization algorithm for 30 trials in different work zones.

	Average Time Taken (in Milli Seconds)	Standard Deviation (in Milli Seconds)	Maximum Time Needed with 95% CI (in Milli Seconds)
Zone-1	12.4704	0.6921	12.7181
Zone-2	12.9060	1.3820	13.4005
Zone-3	11.7888	0.0449	11.8048
Zone-4	11.9322	0.2958	12.0381

**Table 5:** Latency table representing the overall response time of the NCVAD system as a whole.

Activity Description	Responsible Subsystem	Latency Introduced (in Seconds)
ArUco Detection and Object Identification	Environmental Perception Subsystem (EPS)	80.400 × 10^−3^
Audio Prompting (with 1500 ms delay) and Coordinate Calculation	Information Processing and User Prompting Subsystem (IPUPS)	25.301
EEG Data Acquisition and Jaw-Clenching Classification	Neural Command Acquisition Subsystem (NCAS)	0.368
GD-based POA for Coordinate Calculation	Robotic Manipulator Actuation Subsystem (RMAS)	0.424 × 10^−3^
Robotic Arm Actuation for Reach Task	Robotic Manipulator Actuation Subsystem (RMAS)	0.548
Overall Time Taken for All Activities	The Neuronally Controlled Visual Assistive Device (NCVAD)	26.297 *

* The total value is calculated by summing up the latency for individual subsystems.

**Table 6:** Performance of the present methodologies compared to previously established studies and benchmarks.

Author Names	Description for Benchmark		Description for Current Study	
	Methods Used/ Targets Achieved	Obtained Performance Parameters	Methods Used/Targets Achieved	Obtained Performance Parameters
Velez et al. [27]	Neurosky Mindwave headset used for jaw-clenching detection	18 out of 20 correct detections	Emotiv EPOC Flex Headset used for jaw-clenching detection	26 out of 30 correct detections
Masud et al. [63]	P300 event related potential-based EEG data classification	Accuracy achieved: 87.5%	Jaw-clenching trigger-based EEG data classification	Accuracy achieved: 86.67%
Khoshnam et al. [28]	EEG-based control for movement in virtual environment using jaw-clenching trigger	Sensitivity achieved: 80%	EEG-based control for real-time actuation of robotic arm using jaw-clenching trigger	Sensitivity achieved: 96.29%
Akram et al. [64]	P300 event-related potential-based paradigm for intelligent home control	Time taken for selection process: 31.5 s	Jaw-clenching-based EEG paradigm for assistive robotic arm control	Average time taken for all processes: 26.3 s

**Table 7:** Comparison between the implemented user evaluation metrics to the definition presented by ISO 9241-11.

User Evaluation Metrics	Definition as per ISO 9241-11	Corresponding Implementation in the Current Study
Effectiveness	Accuracy and completeness in accomplishing tasks	Selection accuracies and percentage calculations per trial
Efficiency	Resources expended relative to task goals	Task completion time and reaction latency evaluation
Satisfaction	User comfort and acceptability	Post-session questionnaires for user feedback on system efficacy

**Table 8:** Raw and scaled NASA-TLX score for the participant’s response.

S. No.	Subscale	Raw Score by Participant (0–20)	Scaled Score (0–100)	Notes and Remarks
1	Mental Demand: How mentally demanding was the task?	8	40	Somewhat low mental demand
2	Physical Demand: How physically demanding was the task?	2	10	Very low physical demand
3	Temporal Demand: How hurried or rushed was the pace of the task?	11	55	Moderate temporal demand
4	* Performance: How successful were you in accomplishing what you were asked to do?	3	15	Participants perceive high success rate
5	Effort: How hard did you have to work to accomplish your level of performance?	4	20	Low effort by participant
6	Frustration: How insecure, discouraged, irritated, stressed, and annoyed were you?	1	10	Very low frustration reported by the participant (except for mild under confidence during the first trial of the experiment)

* The performance scale is reversed (from perfect to failure) contrary to the other subscales (very low to very high).

**Table 9:** Participant’s response to System Usability Study (SUS) questionnaire.

S. No.	Questionnaire	User Response				
		Strongly Disagree (1)	Disagree (2)	Neutral (3)	Agree (4)	Strongly Agree (5)
1	I think that I would like to use this system frequently.				×	
2	I found the system unnecessarily complex.	×				
3	I thought the system was easy to use.					×
4	I think that I would need the support of a technical person to be able to use this system.			×		
5	I found the various functions in this system were well integrated.					×
6	I thought there was too much inconsistency in this system.	×				
7	I would imagine that most people would learn to use this system very quickly.				×	
8	I found the system very cumbersome to use.		×			
9	I felt very confident using the system.			×		
10	I needed to learn a lot of things before I could start working with this system.	×				

## Data Availability

The original contributions presented in this study are included in the article. Further inquiries can be directed to the corresponding author(s).

## Abbreviations

The following abbreviations are used in this manuscript:

VAD	Visual Assistive Device
NCVAD	Neuronally Controlled Visual Assistive Device
EEG	Electroencephalogram
BCI	Brain–Computer Interface
ERP	Event Related Potential
SSVEP	Steady State Visually Evoked Potential
YOLO	You Only Look Once
YOLOv8n	You Only Look Once, version 8, nano variant
mAP	Mean Average Precision
ArUco	Augmented Reality University of Cordoba
MATLAB	Matrix Laboratory
GD	Gradient Descent
POA	Path Optimization Algorithm
CV	Computer Vision
EPS	Environmental Perception Subsystem
IPUPS	Information Processing and User Prompting Subsystem
NCAS	Neural Command Acquisition Subsystem
RMAS	Robotic Manipulator Actuation Subsystem
CPU	Central Processing Unit
GPU	Graphics Processing Unit
ONNX	Open Neural Network Exchange
TensorRT	Tensor Runtime
FLOPs	Floating Point Operations per Second
COCO	Common Objects in Context
TTS	Text-to-Speech
WPM	Words per Minute
PnP	Perspective-n-Point
FBP	Frequency Band Power
LSL	Lab Streaming Layer
SPS	Samples per Second
CMS	Common Mode Sense
DRL	Driven Right Leg
SNR	Signal-to-Noise Ratio
EMG	Electromyogram
EOG	Electro-oculogram
ICA	Independent Component Analysis
SCG	Selection Command Generation
SSD	Selection Statement Dictation
SCDC	Selection Command Detection Classifier
IID	Independently and Identically Distributed
CoV	Coefficient of Variation
AdaBoost	Adaptive Boosting
LR	Learning Rate
TP	True Positive
TN	True Negative
FP	False Positive
FN	False Negative
ANN	Artificial Neural Network
ReLU	Rectified Linear Unit
DoF	Degrees of Freedom
VDC	Volts Direct Current
EE	End Effector
IK	Inverse Kinematics
DH	Denavit-Hartenberg
SSE	Sum of Squared Error
Z-*i*	Zone-*i*
P-*i*	Participant-*i*
CI	Confidence Interval
IQR	Inter-Quartile Range
TBS	Tactile Blind Spots
TLX	Task Load Index
SUS	System Usability Score
